# Tumoral Interferon Beta Induces an Immune-Stimulatory Phenotype in Tumor-Associated Macrophages in Melanoma Brain Metastases

**DOI:** 10.1158/2767-9764.CRC-24-0024

**Published:** 2024-08-21

**Authors:** Julia Gellert, Dennis A. Agardy, Swaminathan Kumar, Alexandros Kourtesakis, Tamara Boschert, Kristine Jähne, Michael O. Breckwoldt, Lukas Bunse, Wolfgang Wick, Michael A. Davies, Michael Platten, Theresa Bunse

**Affiliations:** 1 German Cancer Research Center (DKFZ) Heidelberg, Clinical Cooperation Unit (CCU) Neuroimmunology and Brain Tumor Immunology, Heidelberg, Germany.; 2 German Cancer Consortium (DKTK), DKFZ, Core Center Heidelberg, Heidelberg, Germany.; 3 Department of Neurology, Medical Faculty Mannheim, Mannheim Center for Translational Neuroscience (MCTN), Heidelberg University, Mannheim, Germany.; 4 Faculty of Bioscience, Heidelberg University, Heidelberg, Germany.; 5 Department of Melanoma Medical Oncology, The University of Texas MD Anderson Cancer Center, Houston, Texas.; 6 German Cancer Research Center (DKFZ) Heidelberg, Clinical Cooperation Unit (CCU) Neurooncology, Heidelberg, Germany.; 7 Neurology Clinic and National Center for Tumor Diseases, University Hospital Heidelberg, Heidelberg, Germany.; 8 Helmholtz Institute for Translational Oncology Mainz (HI-TRON Mainz)—A Helmholtz Institute of the DKFZ, Mainz, Germany.; 9 Department of Neuroradiology, Heidelberg University Hospital, Heidelberg University, Heidelberg, Germany.; 10 DKFZ Hector Cancer Institute at the University Medical Center Mannheim, Mannheim, Germany.; 11 Hertie Network of Excellence in Clinical Neuroscience, Frankfurt, Germany.; 12 Immune Monitoring Unit, National Center for Tumor Diseases (NCT), NCT Heidelberg, a partnership between DKFZ and University Hospital Heidelberg, Heidelberg, Germany.

## Abstract

Type I interferons (IFN) are immune-stimulatory cytokines involved in antiviral and antitumor immune responses. They enhance the efficacy of immunogenic anticancer therapies such as radiotherapy by activating both innate and adaptive immune cells. Macrophages are one of the most abundant innate immune cells in the immune microenvironment of melanoma brain metastases (MBM) and can exert potent immune-suppressive functions. Here, we investigate the potential of tumoral type I IFNs to repolarize tumor-associated macrophages (TAM) in two murine MBM models and assess the effects of radiotherapy-induced type I IFN on TAMs in a transcriptomic MBM patient dataset. In mice, we describe a proinflammatory M1-like TAM phenotype induced by tumoral IFNβ and identify a myeloid type I IFN-response signature associated with a high M1/M2-like TAM ratio. Following irradiation, patients with MBM displaying a myeloid type I IFN-response signature showed increased overall survival, providing evidence that tumoral IFNβ supports an effective antitumor immune response by re-educating immune-regulatory TAM. These findings uncover type I IFN–inducing therapies as a potential macrophage-targeting therapeutic approach and provide a rationale for combining radiotherapy with concomitant immunotherapy to improve treatment response in patients with MBM.

**Significance:**

Our study shows that re-education of tumor-associated macrophages by tumoral IFNβ translates into improved clinical outcome in patients with melanoma brain metastases, providing pathomechanistic insights into synergistic type I interferon–inducing therapies with immunotherapies and warranting investigation of IFNβ as a predictive biomarker for combined radioimmunotherapy.

## Introduction

Type I interferons (IFN) are proinflammatory cytokines known for their role in antiviral immune responses (1). Increasing evidence indicates that the success of several anticancer therapies, including radiotherapy, chemotherapy and immunotherapy, rely on the immune-stimulatory effects of type I IFN on the tumor microenvironment (TME; refs. 2–5).

Type I IFN comprise IFNα, IFNβ, and the less-studied IFNε, IFNκ, and IFNω (6). Type I IFN are secreted by many cell types, mainly immune cells such as dendritic cells (DC) and macrophages and also stromal cells like fibroblasts and endothelial cells (7). They are primarily induced following the stimulation of pattern recognition receptors (PRR) by viral and bacterial components called pathogen-associated molecular patterns (PAMP). In the context of cancer, tumor cell–derived damage-associated molecular patterns (DAMP) such as nucleic acids that are released into the cytosol and the extracellular space during immunogenic anticancer therapies also elicit strong type I IFN responses (8). Activated intracellular PRRs like the cytoplasmic DNA sensor cyclic GMP-AMP synthase (cGAS) or endosomal toll-like receptors (TLR) then signal via stimulator of IFN genes protein (STING) or TNF receptor–associated factors (TRAF) to induce the IFN regulatory factor 3 (IRF3)- or IRF7-dependent transcription of type I IFN-coding genes, respectively (9).

Upon secretion, type I IFN bind to the heterodimeric transmembrane IFNα/β receptor (IFNAR1-IFNAR2) on various immune cell subsets such as macrophages, DCs and T cells and activate the transcription of IFN-stimulated genes (ISG) in a paracrine manner (10). Additionally, autocrine signaling can augment type I IFN responses (11). ISG encode many proteins that exert various immune-stimulatory effects on the TME, thereby promoting cancer immunosurveillance and regression. ISG encoding chemo-attractants like CXC-motif chemokine 10 (CXCL10) lead to an increase in tumor-infiltrating leukocytes (TIL; ref. 12) and by stimulating DC maturation and enhancing their capacity to process and present tumor cell–associated antigens, ISG promote TIL priming and activation (13). Upregulation of ISG encoding for major histocompatibility complex (MHC) class I expression further support TIL-mediated tumor cell killing (13). During inflammation, macrophage-specific ISG enhance macrophage responsiveness to pathogen exposure by boosting their phagocytotic and antigen-presenting capacities (14). In tumor-associated macrophages (TAM), autocrine type I IFN signaling sustains a proinflammatory M1-like phenotype (15) and downregulation of IFNAR1 has been shown to promote immune-suppressive activity in tumor-infiltrating myeloid cells (16). TAM can be classified into immune-stimulatory M1-like (classically activated, antitumor) and immune-regulatory M2-like (alternatively activated, protumor) polarization states (17). However, macrophage polarization states are highly dynamic and M1-like TAM readily acquire a M2-like phenotype when exposed to immune-suppressive cytokines like interleukin 4 (IL4), IL10, transforming growth factor β (TGFβ) and to hypoxia, conditions associated with tumor growth and progression (17). A high degree of macrophage infiltration and a low M1/M2-like ratio correlate with poor patient prognosis in many solid cancers (18). TAM make up the largest immune cell population in primary melanomas (19) and analyses of melanoma brain metastases (MBM) revealed that TAM also substantially contribute to the immune microenvironment of intracranial melanoma metastases, accounting for up to 80% of infiltrating immune cells (20, 21). Different therapeutic approaches of TAM repolarization as part of an effective anticancer therapy are being investigated (22). Although the cellular effects of type I IFN on macrophages during infection are well-studied, not much is known about potential proinflammatory re-educative effects on immunosuppressive M2-like TAM.

In this study, we investigate whether tumor cell-derived type I IFN can re-educate TAM in MBM and thereby foster the efficacy of immunogenic anticancer therapies. Type I IFN-inducing anticancer therapies such as irradiation are characterized by the induction of a plethora of intracellular and intercellular pathways within the TME. Tumor cell-associated antigen release and secretion of other cytokines act immune-stimulatory. To distinguish direct type I IFN–mediated effects from these confounding factors, we develop inducible IFNβ-overexpressing mouse models based on two different melanoma cell lines. We demonstrate that in the absence of immunogenic cell death, tumoral IFNβ is sufficient to elicit an antitumor immune response characterized by re-educating TAM toward a proinflammatory M1-like phenotype and tumor regression in two MBM mouse models. Further, we report a myeloid type I IFN-response signature that is associated with an increased M1/M2-like TAM ratio and increased overall survival in MBM patients treated with radiotherapy.

## Materials and Methods

### Cell lines and lentiviral transduction

YUMM5.2 (Cat# CRL-3367, RRID# CVCL_JK43, sex = male) and B16-F10 (Cat# CRL-6475, RRID# CVCL_0159, sex = male) cells were purchased from the American Tissue Type Collection (ATCC) and cultured in Dulbecco’s Modified Eagle Medium (DMEM) Nutrient Mixture F12 (DMEM/F-12, ThermoFisher Cat# 11320033) supplemented with 10% fetal bovine serum (FBS, ThermoFisher Cat# A5670701), 100 U/mL penicillin (ThermoFisher Cat# 15070063), 0.1 mg/mL streptomycin (ThermoFisher Cat# 15070063) and 0.1 mmol/L non-essential amino acids (ThermoFisher Cat# 11140050) or in DMEM (ThermoFisher Cat# 11965092) supplemented with 10% FBS, 100 U/mL penicillin and 0.1 mg/mL streptomycin, respectively, at 37°C, 5% CO_2_. Cell lines were passaged no more than 10 times after collection or thawing. Every 3 months, cells were tested negative for mycoplasma using the Mycoplasma-PCR Venor GeM Classic Kit (MB Minerva Biolabs Cat# 11-1100). For lentiviral transduction, DNA was isolated from spleen tissue of healthy donor C57BL/6J mice (RRID# MGI:3028467) using the DNeasy Blood&Tissue Kit (Qiagen Cat# 69506) and the *Ifnb1* gene was amplified using the CloneAmp HiFi PCR Premix (TakaraBio Cat# 639298). *Ifnb1* primers are listed in Supplementary Table S1. The PCR product was purified using an E-Gel EX 4% Agarose (Invitrogen Cat# G401004) and BsaI and BsaII restriction sites were attached. A pENTRY Ifnb1-P2A-eGFP vector was created using the NEBridge Golden Gate Assembly Kit BsaI-HF v2 (New England Biolabs Cat# E1601S) and inserted into the pCW57.1 destination vector (RRID# Addgene_41393, Supplementary Fig. S1A) kindly provided by Stefan Pusch [DKTK Clinical Cooperation Unit Neuropathology, German Cancer Research Center (DKFZ), Heidelberg, Germany] using the Gateway Cloning System (ThermoFisher) and into the LeGO-T2 vector (RRID# Addgene_27342, Supplementary Fig. S1B) kindly provided by Boris Fehse using the In-Fusion Snap Assembly Master Mix (Takara Cat# 638947) for transduction of the YUMM5.2 and B16-F10 cell lines, respectively. The final *Ifnb1*-P2A-*eGFP* cassette containing a tetracycline response promotor p_thight_ followed by the minimal CMV promotor (Supplementary Fig. S1A and S1B) allowed a Doxycycline (Dox)-dependent transcription of *Ifnb1* and *eGFP.* Additionally, the hPGK promoter allowed constitutive expression of the puromycin resistance gene enabling puromycin-based cell selection. To compensate for potentially increased immunogenicity caused by Dox-dependent eGFP co-expression, a P2A-*eGFP* control vector was generated. All vectors generated in this study are available upon request from the corresponding author. Lenti-X 293T cells (TakaraBio Cat# 632180, RRID# CVCL_4401) or HEK293 cells (ATCC Cat# CRL-1573, RRID# CVCL_0045) were transfected with pCW Ifnb1-P2A-eGFP, pCW P2A-eGFP and pLeGO-T2-Ifnb1-P2A-eGFP using the Ecotropic Lenti-X Packaging Single Shots (TakaraBio Cat# 631278) or second generation packaging plasmids psPAX2 (RRID# Addgene_12260) and pMD2.G (RRID# Addgene_12259) kindly provided by Didier Trono with the TransIT-LT1 Transfection Reagent (Mirus Bio Cat# MIR 2304), respectively. Lentiviral supernatant was collected 24 and 48 hours after transfection, spun down to remove residual cells, filtered and used to transduce YUMM5.2 or B16-F10 cells in the presence of 8 μg/mL polybrene (Sigma-Aldrich Cat# TR-1003-G). Successfully transduced cells were selected with medium supplemented with 2.5 μg/mL (YUMM5.2 Ifnb1eGFP and YUMM5.2 eGFP) or 5 μg/mL (B16-F10 Ifnb1eGFP) puromycin (ThermoFisher #Cat A1113803) followed by fluorescence-activated cell sorting of eGFP positive cells (Supplementary Fig. S1C). For *eGFP*- and *Ifnb1*-induction, medium was supplemented with 1 μg/mL of doxycycline hyclate (Dox, Sigma-Aldrich #Cat D9891). YUMM5.2 Ifnb1eGFP and YUMM5.2 eGFP were cultured in DMEM/F-12 supplemented with 10% FBS, 100 U/mL penicillin, 0.1 mg/mL streptomycin, 0.1 mmol/L non-essential amino acid and 0.25 μg/mL puromycin at 37°C, 5% CO_2_. B16-F10 Ifnb1eGFP and B16-F10 eGFP were cultured in DMEM supplemented with 10% FBS, 100 U/mL penicillin, 0.1 mg/mL streptomycin and 0.5 μg/mL puromycin at 37°C, 5% CO_2_. All kits and components were used according to the manufacturer’s instructions.

### Animal experiments

Female C575BL/6J (RRID# MGI:3028467) and Balb/cN (RRID# MGI:2161229) wild-type mice were purchased from Janvier Labs at the age of 10 to 12 weeks and allowed to adjust for 7 days. Animal procedures followed institutional laboratory animal research guidelines and were performed under license numbers G170-21, G11-22, and DKFZ268 approved by governmental authorities (Regional Administrative Authority Karlsruhe, Germany). A total of 5,000 YUMM5.2 Ifnb1eGFP, YUMM5.2 eGFP or B16-F10 Ifnb1eGFP cells were stereotactically injected into the right hemisphere (coordinates: 2 mm lateral of the bregma, 1 mm anterior to the coronal suture and 3 mm below the dura) using a 10 μL Hamilton micro-syringe (Hamilton Company Cat# 80350) driven by a fine step stereotactic device (Stoelting RRID# SCR_025303) at a concentration of 5,000 cells/μL PBS. The borehole was sealed using bone wax (Ethicon Cat# W31G). Tumor cell inoculation was performed under anesthesia and mice were treated with analgesics for 48 hours post-surgery. Mice were checked daily for tumor-related symptoms and sacrificed when mice showed signs of neurological deficit or stop criteria were met. For tumoral IFNβ induction, mice were treated with diet containing 625 mg/kg Dox (Ssniff Cat# A112D00623). Tumor growth and treatment response was monitored by MRI on day 14 post inoculation at the Small Animal Imaging Core Facility, German Cancer Research Center (DKFZ, RRID# SCR_025301) using a Bruker BioSpec 3Tesla (RRID# SCR_025302) or at the Radiology Department, University Clinic Heidelberg, using a Bruker BioSpec 94/20 (RRID# SCR_018054) with a four-channel phased-array surface receiver coil. Tumor volumetry was based on T1 and T2 weighted images after contrast agent application and was determined by manual segmentation using Bruker ParaVision software 360 (v6.0.1, RRID# SCR_025295) or 3D Slicer software (v4.11.20210226, RRID# SCR_005619).

### Generation and treatment of bone marrow–derived macrophages

Bone marrow cells were extracted from hip bones, femur and tibia from C575BL/6J donor mice sacrificed by cervical dislocation by grinding the bones in IMDM medium (ThermoFisher Cat# 12440053). Cells were mashed through a 40 μm strainer and erythrocytes lysed using ACK lysing buffer (ThermoFisher Cat# A1049201). Bone marrow cells were seeded at a concentration of 1 × 10^6^ cells/mL IMDM medium supplemented with 10% FBS, 100 U/mL penicillin, 0.1 mg/mL streptomycin and 20 ng/mL M-CSF (Peprotech Cat# 315-02). After 72 hours, half of the medium supplemented with M-CSF was replaced. After 96 hours, *in vitro* differentiated macrophages were harvested and seeded at a concentration of 1.7 × 10^5^ cells/cm^2^. Bone marrow–derived macrophages (BMDM) were polarized into M1-like or M2-like macrophages by supplementing BMDM medium with 1 ng/mL IFNγ (Peprotech Cat# 315-05) and 2.5 ng/mL LPS (Sigma-Aldrich #L5293) or with 10 ng/mL IL4 (Peprotech Cat# 214-14), respectively. After 48 hours, BMDM were treated with BMDM medium supplemented with recombinant IFNβ (Bio-Techne Cat# 8234-MB-010), tumor cell culture supernatant with tumoral IFNβ, tumor cell culture supernatant or unsupplemented BMDM medium as control. Cell culture supernatant was obtained from YUMM5.2 Ifnb1eGFP or B16-F10 Ifnb1eGFP cells after 72 hours of treatment with 1 μg/mL Dox. IFNβ concentration in tumor cell culture supernatant was measured with ELISA as described below and supernatant was diluted with BMDM medium to a concentration of 1,500 pg/mL IFNβ. Recombinant IFNβ was diluted with BMDM medium to the respective concentrations. Supernatant obtained from YUMM5.2 and B16-F10 control cells was diluted at the same ratio as tumor cell supernatants with tumoral IFNβ. BMDMs were cultured for 72 hours before cells were harvested for flow cytometry analysis.

### Mixed leukocyte reaction

BMDM were generated, polarized and pretreated as described above. After 72 hours of IFNβ pretreatment, T cells were extracted from spleens and lymph nodes from Balb/cN donor mice sacrificed by cervical dislocation. Cells were mashed through a 70 μm strainer and erythrocytes lysed using ACK lysing buffer. Lymphocytes were enriched for CD3^+^ T cells using the Pan T Cell Isolation Kit II (Miltenyi BioTech Cat# 130-095-130) and subsequently stained using the CellTrace Far Red Proliferation Kit (ThermoFisher Cat# C34564, RRID# AB_3097723) at a 1 μmol/L staining solution according to the manufacturer’s instructions. T cells were added to BMDM at BMDM to T-cell ratios of 1:1 and 1:10 in RPMI 1640 (PAN-Biotech Cat# P04-18500) supplemented with 10% FBS, 100 U/mL penicillin, 0.1 mg/mL streptomycin, 2 mmol/L L-glutamine (Sigma-Aldrich Cat# G5792), 25 mmol/L HEPES pH 7.4 (ThermoFisher Cat# J67485.AK), 1 mmol/L sodium pyruvate (Sigma-Aldrich Cat# S8636), 0.1 mmol/L NEAA (Sigma-Aldrich Cat# M7145) and 5 × 10^−5^ mol/L β-mercaptoethanol (ThermoFisher Cat# 31350010). After 5 days, T cells were harvested for flow cytometry analysis of T-cell proliferation by CTFR dye dilution and of CD4^+^ and CD8^+^ T-cell phenotypes. Unstained CTFR T cells and stained CTFR T cells activated using Dynabeads Mouse-T-Activator CD3/CD28 (ThermoFisher Cat# 11456D) were used as proliferation controls.

### Cell proliferation assay

Tumor cells were stained using the CellTrace Far Red Proliferation Kit at a 0.5 μmol/L staining solution according to the manufacturer’s instructions and subsequently seeded at a concentration of 2.6 cells/cm^2^. After 24 hours, cells were treated with medium supplemented with 1 μg/mL Dox. Tumor cells were cultured for 48 hours before cells were harvested for flow cytometry analysis of cell proliferation by CTFR dye dilution. Unstimulated tumor cells were used as proliferation controls.

### Flow cytometry

For *ex vivo* investigation of YUMM5.2 Ifnb1eGFP, YUMM5.2 eGFP and B16-F10 Ifnb1eGFP brain tumors, mice were perfused with ice-cold PBS (Sigma-Aldrich Cat# D8537) and the right tumor-bearing hemisphere was excised. Tumor homogenates were generated by mechanical dissection followed by enzymatic digestion of the right tumor-bearing hemisphere in HBSS (Sigma-Aldrich Cat# H99269) supplemented with 50 μg/mL Liberase (Roche Cat# 05989132001) for 30 minutes at 37°C. Tumor homogenates were centrifuged and supernatant was collected for ELISA (see below). Tumor cell suspensions were mashed through 100 and 70 μm cell strainers and myelin was removed with a 30% continuous Percoll (Sigma-Aldrich Cat# GE17-0891-01) gradient. Cells were blocked with anti-CD16/CD32 antibody (ThermoFisher Cat# 14-0161-82, RRID# AB_467133) for 5 minutes at room temperature. Extracellular targets were stained in PBS supplemented with antibodies (100 μL/1 × 10^6^ cells) for 30 minutes at 4°C in the dark. For intracellular staining, cells were fixed, permeabilized and stained using eBioscience Foxp3/Transcription Factor Staining Buffer Set (ThermoFisher Cat# 00-5523-00) for 45 minutes at 4°C in the dark. Antibodies are listed in Supplementary Table S2. Unfixed samples were acquired within 4 hours and fixed samples within 48 hours using a BD FACS Canto II (BD Biosciences, RRID# SCR_018056), a BD LSR Fortessa (BD Biosciences, RRID# SCR_018655) or a ZE5 Cell Analyzer (Bio-Rad, RRID# SCR_019712). Gating and data analysis were performed using FlowJo software (v10.4, RRID# SCR_008520). Gating strategies are shown in Supplementary Figs. S2 and S3G.

### IFNβ ELISA

IFNβ concentration of cell culture supernatants and tumor homogenates or plasma was determined using the standard or high sensitivity Mouse IFN Beta ELISA Kit (pbl Assay Science Cat# 42400 and 42410), respectively. Tumor homogenates were generated as described above and IFNβ concentrations normalized to tumor volume. All kits and components were used according to the manufacturer’s instructions.

### Immunohistochemistry

Mice were perfused with ice-cold PBS and excised brains were fixed using ROTIHistofix 4% (Roth Cat# P087.4) for 24 hours at 4°C. Brains were transferred to 30% sucrose (ThermoFisher Cat# J65148.A1) solution and incubated for 48 hours at 4°C. Brains were embedded in cryomolds in Tissue-Tek O.C.T. (Sakura Cat# SA62550-01) and frozen at −80°C. Sections were air dried for 30 minutes and subsequently fixed for 10 minutes with methanol (ThermoFisher Cat# L13255.AP) on ice. Blocking buffer consisting of PBS supplemented with 0.1% TWEEN 20 (Sigma-Aldrich Cat# 11332465001) and 4% of the appropriate serum was applied and sections were incubated for 1 hour at room temperature. Co-staining was performed with primary antibodies diluted in blocking buffer overnight at 4°C either simultaneously (MHCI, CD206 and CD86) or sequentially (CD69). Antibodies are listed in Supplementary Table S2. Sections were washed with PBS supplemented with 0.1% TWEEN 20. Secondary antibodies were diluted in blocking buffer and applied for 1 hour at room temperature. Sections were washed and mounted using Fluoromount-GTM with DAPI (Thermo Fisher Cat# 00-4959-52). Images were acquired within 72 hours on a LSM700 confocal microscope (Zeiss, RRID# SCR_017377) using a 20× objective and consistent laser intensity, gain and image settings. Image acquisition was restricted to representative areas within the tumor as per macroscopic evaluation. For graphical representation, brightness and contrast of original images was adjusted using Fiji (v2.14.0, RRID# SCR_002285) and applied to all merged and single-channel images uniformly. For quantification, unprocessed images were analyzed using Fiji.

### qRT-PCR

Single cell suspensions from YUMM5.2 Ifnb1eGFP brain tumors were generated as described above and enriched for F4/80+ TAM using the Anti-F4/80 MicroBeads UltraPure (Miltenyi BioTech Cat# 130-110-443). Total RNA was isolated using the RNeasy Mini Kit (Qiagen Cat# 74104) and on-column DNase digestion using the RNAse-Free DNase Set (Qiagen Cat# 79254) was performed. RNA concentration was measured with NanoDrop ND-1000 (ThermoFisher, RRID# SCR_016517). cDNA was synthesized using the High-Capacity cDNA Reverse Transcription Kit (Applied Biosystems Cat# 4368814). RT-qPCR was performed in technical triplicates per biological replicate using the 2x qPCR SYBRGreen Master Mix high ROX (Steinbrenner Cat# SL-9902HR) on a QuantStudio 3 Real Time PCR System (ThermoFisher, RRID# SCR_018712). Primers are listed in Supplementary Table S1. CT values were normalized to the house-keeping gene *Gapdh* and analyzed using the 2−∆∆CT method. All kits and components were used according to the manufacturer’s instructions.

### Bulk RNA sequencing and analyses

BMDM were generated, polarized, pretreated and total RNA was isolated as described above. Samples passed quality control if RNA Integrity Number (RIN) measured using the Agilent 4200 TapeStation System (Agilent, RRID# SCR_018435) was between 8 and 10. Libraries were prepared using the TruSeq Stranded Total RNA Kit (Illumina) and sequenced with the NextSeq 500/550 High Output Kit v2.5 (Illumina) at the NGS Core Facility, German Cancer Research Center (DKFZ, RRID# SCR_012503). GRCm39 mm10 was used as a reference genome for RSEM (v1.3.3, RRID# SCR_000262) alignment. Gene count estimates were then imported into R (v4.1.0, RRID# SCR_001905). Count matrix was normalized and differential gene expression analysis was performed using DESeq2 (v1.32, RRID# SCR_015687; ref. 23) with log_2_ fold change ≥2 and adjusted *P* value ≤ 0.01.

### Patient bulk RNA sequencing data analysis

Raw sequencing reads from patients with MBM were generated and analyzed as described previously under an institutionally approved protocol (23). Differential gene expression analysis was performed using DESeq2 (24) with log_2_ fold change ≥1 and adjusted *P* value ≤ 0.05. Gene set enrichment analysis (GSEA) was conducted using clusterProfiler (v4.0.5, RRID# SCR_016884). All kits and components were used according to the manufacturer’s instructions.

### Statistics

Sample sizes of six and eight animals were defined according to the primary outcome of TME analysis and type I IFN quantification or tumor growth, respectively. Animals were randomized to either control or Dox treatment. Tumor growth and treatment response was monitored by MRI on day 14 post-tumor cell inoculation and MR images were evaluated for tumor volume by blinded observers. Animals with no distinguishable tumor were excluded from tumor growth analyses. Animals that were symptomatic before the predefined endpoint were excluded from all subsequent analyses. Group sizes (*n*) and applied statistical tests are indicated in the figure legends. Statistical tests were performed in R using rstatix (v0.7.2, RRID# SCR_021240). Significance was assessed by either paired *t* test for normally distributed variables, Mann–Whitney *U* test for not-normally distributed variables, one-way analysis of variance (ANOVA) with Tukey *post hoc* testing or log-rank test following Kaplan–Meier survival estimates as indicated in the figure legends. Effect sizes of 0.10 to < 0.3 were considered as small effect, 0.30 to < 0.5 as moderate effect and ≥0.5 as large effect. Spearman correlation was applied for correlation analysis. All data are presented as individual values, as median ± IQR with individual values or as mean.

### Data availability

The data generated in this study are available within the article and its supplementary files. BMDM bulk RNA sequencing data were deposited in the GEO repository and are available under accession number GSE271267. RNA sequencing data of patients with MBMs were obtained via the European Genome-Phenome Archive (EGA; accession number EGAS00001003672) under license by the authors.

## Results

### Tumoral IFNβ suppresses intracranial tumor growth of murine YUMM5.2 melanoma cells

**Figure 1:**
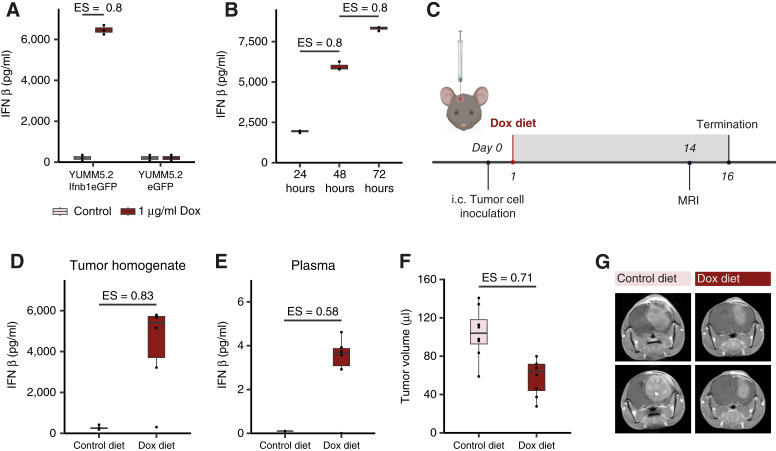
Induction of tumoral IFNβ impairs intracranial YUMM5.2 Ifnb1eGFP tumor growth. **A,** YUMM5.2 Ifnb1eGFP and YUMM5.2 eGFP control cells were treated with 1 μg/mL Dox or left untreated for 48 hours before cell culture supernatant was harvested. IFNβ concentrations were analyzed by ELISA and are displayed in pg/mL (*n* = 3). **B,** YUMM5.2 Ifnb1eGFP cells were treated with 1 μg/mL Dox and cell culture supernatant was harvested after 24, 48, and 72 hours. IFNβ concentrations were analyzed by ELISA and are displayed in pg/mL (*n* = 3). **C–G,** C57Bl/6J mice received control diet or Dox diet from day 1 on following intracranial YUMM5.2 Ifnb1eGFP tumor cell inoculation. Tumor growth was monitored by MRI on day 14, tumors were excised and blood was drawn on day 16. **C,** Schematic timeline. **D,** Intratumoral IFNβ concentrations were analyzed by ELISA and are displayed in pg/mL of original tumor volume (*n* = 6 for each group). **E,** Plasma IFNβ concentrations were analyzed by ELISA and are displayed in pg/mL. (*n* = 6 for each group). **F,** Tumor volume as measured by MRI is displayed in μL (T1 sequence; *n* = 8 for each group). **G,** Representative coronal MR images in T1 sequence with contrast agent. Each image represents one mouse from the indicated treatment group. Data are expressed as median + IQR and individual values for **A**, **B**, and **D–F**. Statistical significance was determined by Mann–Whitney *U* test for **A**, **B**, and **D–F**. ES, effect size. (**C**, Created with BioRender.com.)

To study the direct effects of type I IFN on the immune microenvironment of MBM without the confounding effects of immunogenic cell death and other immune-stimulatory cytokines released during type I IFN–inducing anticancer therapies such as radiation, we first generated a Tet-On inducible IFNβ-overexpressing mouse model using the YUMM5.2 melanoma cell line. YUMM5.2 cells are derived from a murine melanoma driven by Braf activation and p53 inactivation, both frequently occurring oncogenic mutations in cutaneous melanoma as well as MBM (25, 26). We chose to overexpress IFNβ because this member of the type I IFN family can be produced by stromal cells like fibroblasts and endothelial cells as well as macrophages, while IFNα is primarily produced by dendritic cells (11), a rare cell type in the brain parenchyma. We included an *eGFP* tag in the lentiviral construct to sort and select successfully transduced YUMM5.2 Ifnb1eGFP as well as YUMM5.2 eGFP control cells lacking *Ifnb1* (Supplementary Fig. S1A–S1C). Following *in vitro* Dox treatment, YUMM5.2 Ifnb1eGFP cells showed significant IFNβ secretion compared to untreated YUMM5.2 Ifnb1eGFP and Dox-treated YUMM5.2 eGFP control cells (Fig. 1A). IFNβ was measurable in the cell culture supernatant 24 hours after Dox induction and repeated measures showed an increasing IFNβ concentration at 48 and 72 hours (Fig. 1B). Of note, IFNβ secretion after Dox induction did not alter YUMM5.2 Ifnb1eGFP proliferation compared to proliferation of Dox-treated YUMM5.2 eGFP control cells (Supplementary Figs. S1D and S2A). We next aimed to assess the intracranial growth of YUMM5.2 Ifnb1eGFP cells and the effects of tumoral type I IFN induction on tumor growth by magnetic resonance imaging (MRI; Fig. 1C). YUMM5.2 Ifnb1eGFP-bearing animals started to show neurological symptoms after 16 days. Treatment with Dox diet was confirmed to induce significant IFNβ secretion in YUMM5.2 Ifnb1eGFP brain tumors compared to control diet, leading to high intratumoral Ifnβ concentrations (Fig. 1D). Of note, IFNβ plasma levels in Dox-treated YUMM5.2 Ifnb1eGFP-bearing mice of approx. 4 pg/mL (Fig. 1E) were comparable to concentrations detectable in serum of non-small cell lung cancer (NSCLC) patients with brain metastases after radiotherapy and prior to complete or partial response to combined radiotherapy and immune checkpoint inhibition using ipilimumab, which ranged from approximately 4 to maximum 20 pg/mL (27). This hints toward similar intratumoral levels in our model and in patients. Importantly, Dox diet indeed markedly impaired intracranial YUMM5.2 Ifnb1eGFP tumor growth (Fig. 1F and G).

### Tumoral IFNβ modulates YUMM5.2 MBM–associated macrophage polarization

**Figure 2:**
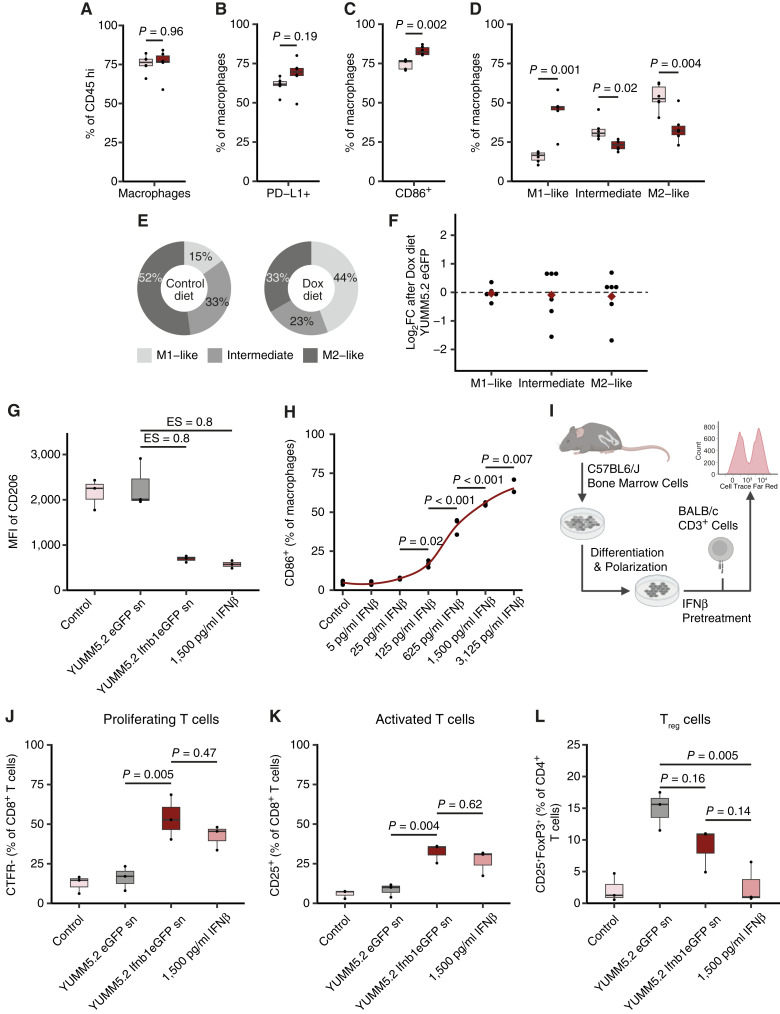
YUMM5.2 Ifnb1eGFP-derived IFNβ modulates macrophage polarization and function. **A–D,** Quantitative flow cytometry analysis of myeloid populations in the YUMM5.2 Ifnb1eGFP TME after control diet (light pink) or Dox diet (dark red). Displayed as percentage of parental population (*n* = 6 for each group). **E,** Ratio of TAM polarization states in the YUMM5.2 Ifnb1eGFP TME after control diet or Dox diet. Displayed as mean percentage per group (*n* = 6 for each group). **F,** Quantitative flow cytometry analysis of TAM polarization states in YUMM5.2 eGFP tumors after Dox diet compared to control diet. Displayed as log_2_ fold change (Log_2_FC; *n* = 6 for each group). **G** and **H,** Quantitative flow cytometry analysis of CD206 (**G**) and CD86 (**H**) in bone marrow-derived and M2-like polarized macrophages after 72 hours of indicated treatments. Displayed as mean fluorescence intensity (MFI) in **G** or percentage of macrophages in **H**. sn, supernatant, collected after Dox pretreatment (*n* = 3). **I–L,** M2-like polarized BMDM generated from C57BL6/J mice were pretreated as indicated and subsequently co-cultured 1:1 (**J **and** K**) or 1:10 (**L**) with CTFR-stained T cells isolated from BALB/c mice. T cells were analyzed on day 5 by flow cytometry. Displayed as percentage of CD8^+^ T cells (**J** and **K**) or of CD4^+^ T cells (**L**). For gating strategies see Supplementary Figs. S2 and S3G. M1-like macrophages were defined as MHCII^+^CD206^−^, intermediate macrophages as MHCII^+^CD206^+^ and M2-like macrophages as MHCII^−^CD206^+^ in **D–F**. Data are expressed as median + IQR and individual values for **A–D**, **G**, and **J–L**, mean for **E** and individual values and mean for **F** and **H**. Statistical significance was determined by paired *t* test for **A–D**, by Mann–Whitney U test for **G** and by one-way analysis of variance (ANOVA) with Tukey *post hoc* testing for **H** and **J–L**. ES, effect size. (**I**, Created with BioRender.com.)

To investigate if tumoral IFNβ shapes the TME, we performed flow cytometry analysis of infiltrating immune cells from established YUMM5.2 Ifnb1eGFP brain tumors after late Dox diet, allowing tumors to grow and yield sufficient tumor-infiltrating cells for individual analysis (Supplementary Figs. S2B, S2C and S3A). Intratumoral IFNβ concentrations were comparable to the ones reached with early Dox treatment (Supplementary Fig. S3B). Although flow cytometry analysis revealed no significant effect of tumoral IFNβ on the abundance of CD45^high^ immune cells, macrophages or on the phenotype of YUMM5.2 Ifnb1eGFP-infiltrating T cells (Fig. 2A; Supplementary Figs. S2B, S2C and S3C), tumoral IFNβ exerted a strong effect on the polarization of YUMM5.2 Ifnb1eGFP-infiltrating macrophages (Fig. 2B–E; Supplementary Fig. S2C). TAM from mice receiving Dox diet showed enhanced PD-L1 expression (Fig. 2B), exhibited a more proinflammatory M1-like phenotype as demonstrated by enhanced CD86 (Fig. 2C) and MHCII surface levels (Fig. 2D and E), while displaying a decreased immune-regulatory M2-like phenotype signified by reduced expression of CD206 (Fig. 2D and E; ref. 17), compared to those from mice receiving control diet. Subsequently, we validated enhanced expression of CD86 and decreased expression of CD206 in mice receiving Dox diet in immunohistochemical analyses (Supplementary Fig. S3D and S3E). Corroborating that this effect was mediated by tumoral IFNβ but not Dox, Dox-induced changes of M1- and M2-like TAM phenotypes could not be observed in YUMM5.2 eGFP brain tumors (Fig. 2F). However, indirect type I IFN signaling via stromal cells or increased recruitment of M1-like TAM to YUMM5.2 Ifnb1eGFP tumors could also account for the observed changes in TAM polarization. To assess direct effects of tumoral type I IFN signaling on macrophage repolarization, we treated BMDM, which were polarized toward an M2-like phenotype (Supplementary Fig. S3F), with cell culture supernatant from Dox-treated YUMM5.2 Ifnb1eGFP cells containing tumoral IFNβ or with recombinant (r) IFNβ of concentrations which TAM encounter within YUMM5.2 Ifnb1eGFP tumors after Dox treatment (Fig. 1D; Supplementary Fig. S3B). Compared to untreated M2-like BMDM or those treated with cell culture supernatant from Dox-treated YUMM5.2 eGFP control cells, IFNβ-treated M2-like BMDM expressed lower surface levels of the anti-inflammatory marker CD206 (Fig. 2G) irrespective of the IFNβ source. We next assessed dose-dependent rIFNβ effects on BMDM using concentrations ranging from those found in NSCLC brain metastases patient serum post radiation (27) to intratumoral levels in the YUMM5.2 Ifnb1eGFP model (Fig. 1D and E). Of note, a more proinflammatory BMDM phenotype was observed starting at low rIFNβ concentrations (Fig. 2H). These results indicate a direct IFNβ-mediated repolarization of both M2-like polarized BMDM and YUMM5.2 Ifnb1eGFP-infiltrating macrophages toward a more immune stimulatory phenotype. Ultimately, cytotoxic T-cell responses drive eradication of MBM. To evaluate the impact of IFNβ-exposed macrophages on T cells, we co-cultured IFNβ-pretreated M2-like polarized BMDM with allogeneic T cells (Fig. 2I; Supplementary Fig. S3G). Remarkably, pretreatment of M2-like polarized BMDM with tumoral or rIFNβ indeed enhanced the immune-stimulatory potential of BMDM as evidenced by an increased proliferation (Fig. 2J) and activation (Fig. 2K) of co-cultured CD8^+^ T cells. Of note, co-culture of BMDM pretreated with YUMM5.2 eGFP control cell culture supernatant led to an increase in regulatory T cells, which was in part rescued by exposure of BMDM to YUMM5.2 Ifnb1eGFP-derived IFNβ (Fig. 2L). Taken together, tumoral IFNβ leads to proinflammatory changes of the TMEs that are associated with a reduction in tumor growth.

### Tumoral IFNβ repolarizes TAM and reduces tumor growth in the B16-F10 MBM model

**Figure 3:**
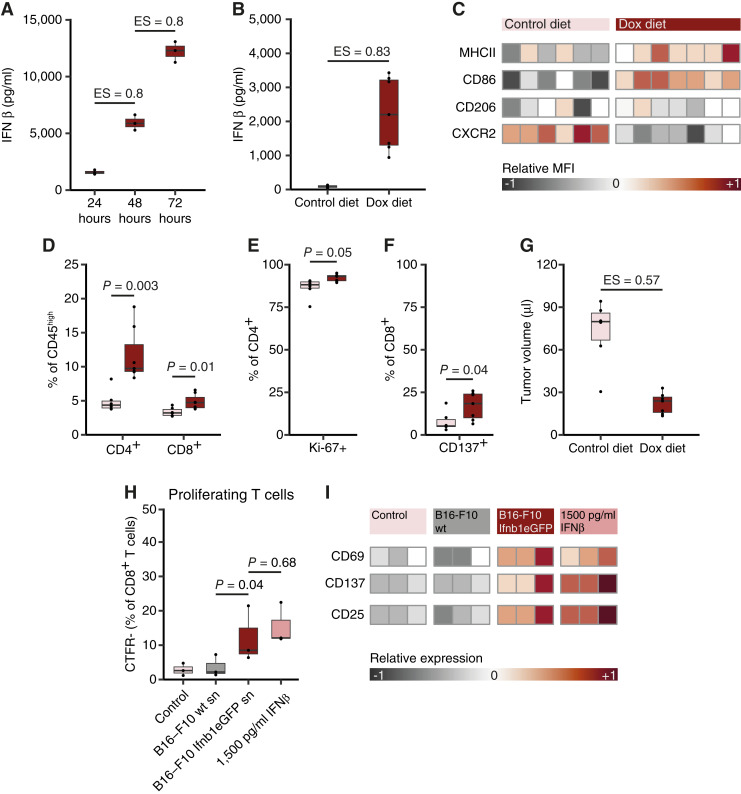
B16-F10 Ifnb1eGFP-derived IFNβ repolarizes macrophages and reduces tumor growth. **A,** B16-F10 Ifnb1eGFP cells were treated with 1 μg/mL Dox and cell culture supernatant was harvested after 24, 48 and 72 hours. IFNβ concentrations were analyzed by ELISA and are displayed in pg/mL (*n* = 3). **B–G,** C57Bl/6J mice received control diet or Dox diet from day 1 on following intracranial B16-F10 Ifnb1eGFP tumor cell injection. Tumor growth was monitored by MRI on day 14, tumors were excised and blood was drawn on day 16. **B,** Intratumoral IFNβ concentrations were analyzed by ELISA and are displayed in pg/mL of original tumor volume (*n* = 6, control diet in light pink; *n* = 7, Dox diet in dark red). **C,** Heatmap of quantitative flow cytometry analysis of B16-F10 Ifnb1eGFP-associated macrophages displayed as relative MFI (*n* = 6 and *n* = 7). **D–F,** Quantitative flow cytometry analysis of B16-F10 Ifnb1eGFP-infiltrating T cells displayed as percentage of parental population (*n* = 6 and *n* = 7). **G,** B16-F10 Ifnb1eGFP tumor volume as measured by MRI is displayed in μL (T1 sequence; *n* = 8). **H** and **I,** Quantitative flow cytometry analysis of CD8^+^ T cells after a 1:1 CTFR-stained T cell to M2-polarized BMDM allo-co-culture after indicated BMDM pretreatment (sn, supernatant collected after Dox pre-treatment; *n* = 3) Displayed as percentage (**H**) or relative expression (**I**) of CD8^+^ T cells. For gating strategies see Supplementary Figs. S2 and S3G. Data are expressed as median + IQR and individual values for **A**, **B**, and **D–H** and individual scaled values for **C** and **I**. Statistical significance was determined by Mann–Whitney U test for **A**, **B**, and **G**, by paired *t* test for **D–F** and by one-way analysis of variance (ANOVA) with Tukey *post hoc* testing for **H**. ES, effect size.

To rule out model specific observations, we next exploited the widely used melanoma cell line B16-F10 for validation of intratumoral education of macrophages by local IFNβ secretion. For this purpose, we genetically modified B16-F10 cells by transduction with an inducible Tet-on Ifnb1eGFP vector and confirmed Dox-induced IFNβ secretion at concentrations similar to YUMM5.2 Ifnb1eGFP (Figs. 1B and 3A; Supplementary Fig. S1B and S1C). Treating B16-F10 Ifnb1eGFP-bearing mice with Dox induced significant intratumoral IFNβ concentrations compared to control diet (Fig. 3B), which were comparable to those in YUMM5.2 Ifnb1eGFP tumors (Fig. 1D) yet did not translate into detectable plasma concentrations (Supplementary Fig. S3H). TME analyses by flow cytometry revealed a more proinflammatory TAM phenotype (Fig. 3C; Supplementary Fig. S3I). While no significant effects on T cells could be observed in YUMM5.2 Ifnb1eGFP brain tumors (Supplementary Fig. S3C), in the B16-F10 model, Dox-induced tumoral IFNβ exhibited significant stimulatory effects on tumor-infiltrating T cells. Both abundance and proliferation of CD4^+^ T cells (Fig. 3D and E) as well as the number and activation of CD8^+^ T cells (Fig. 3D and F) were significantly increased after Dox treatment compared to control treatment. These effects on intratumoral T cells were reflected by clinical response to treatment, with Dox diet significantly reducing intracranial growth of B16-F10 Ifnb1eGFP compared to control treatment (Fig. 3G). Consistent with the *in vivo* B16-F10 TME data and effects of Dox-treated YUMM5.2 Ifnb1eGFP supernatant (Fig. 2J and K), we confirmed an increase in CD8^+^ T-cell proliferation and activation after co-culture with allogenic M2-polarized BMDM that had been pretreated with Dox-induced B16-F10 Ifnb1eGFP supernatant compared to pretreatment with Dox-induced supernatant of control B16-F10 wild-type cells (Fig. 3H and I). Induction of T-cell proliferation was independent of the IFNβ source, with the same frequency of proliferating T cells after co-culture with rIFNβ-treated BMDMs (Fig. 3H). Both treatment of M2-polarized BMDMs with rIFNβ and Dox-induced B16-F10 Ifnb1eGFP supernatant led to increased activation of CD8^+^ T cells after co-culture (Fig. 3I). Collectively, these data confirm a tumoral IFNβ-mediated phenotypic and functional proinflammatory modulation of TAM in the B16-F10 MBM model.

### Transcriptomic analyses of IFNβ-treated macrophages reveal an immune-stimulatory phenotype

**Figure 4:**
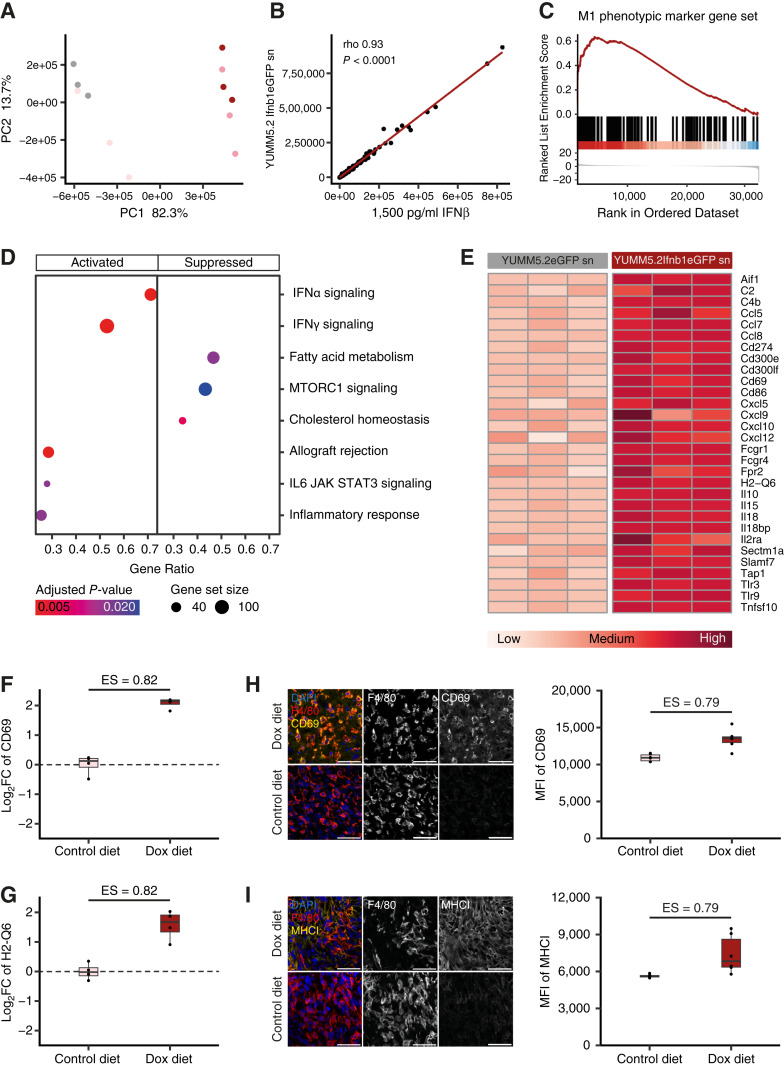
Transcriptomic analyses of IFNβ-treated bone marrow-derived macrophages reveal an immune-stimulatory phenotype characterized by the expression of a proinflammatory type I IFN-response signature. **A,** Principal component analysis of bulk RNA sequencing gene expression profiles of M2-like polarized BMDM treated with supernatant from Dox-treated YUMM5.2 eGFP control cells (gray), supernatant from Dox-treated YUMM5.2 Ifnb1eGFP cells (dark red), rIFNβ (pink) or untreated control (light pink; *n* = 3). **B,** Spearman correlation analysis of log_2_-transformed mean gene counts in M2-like polarized BMDM after indicated treatments. sn, supernatant collected after Dox pre-treatment (*n* = 3). **C,** GSEA of a curated M1 phenotypic marker gene set in M2-like polarized BMDM after treatment with supernatant from Dox-treated YUMM5.2 Ifnb1eGFP cells compared to treatment with supernatant from Dox-treated YUMM5.2 eGFP control cells. Enrichment Score = 0.62, NES = 2.38, adjusted *P* value = 1.00e–10 (*n* = 3). **D,** GSEA of the Molecular Signature Database Hallmark Gene Sets in M2-like polarized BMDM after treatment as in **C**. Top activated and suppressed Hallmark gene sets are displayed according to their gene ratio (*n* = 3). **E,** Heatmap of normalized gene counts of upregulated immune-related genes in M2-like polarized BMDM after indicated treatments (*n* = 3). **F** and **G,** mRNA levels of CD69 (**F**) and H2-Q6 (**G**) measured by qPCR in F4/80+ cells isolated from intracranial YUMM5.2 Ifnb1eGFP tumors from mice receiving control diet compared to Dox diet. Displayed as log_2_ fold change (Log_2_FC; *n* = 4). **H,** Immunohistochemistry staining of F4/80 (red) and CD69 (**H**) or MHCI (**I**; yellow) on tissue sections from mice with intracranial YUMM5.2 Ifnb1eGFP tumors receiving control diet compared to Dox diet. Left, representative images. Scale bars, 50 μm. Right, quantitative analysis of CD69 (**H**) or MHCI (**I**) expression in F4/80+ cells displayed as MFI (*n* = 3, 2 fields of view per animal). For the M1 phenotypic marker gene set see Supplementary Table S3. Data are expressed as individual values for **A**, **B**, **D**, and **E** and median + IQR and individual values for **F**–**I**. Statistical significance was determined by Mann–Whitney *U* test for **F**–**I**. ES, effect size.

To gain a deeper understanding of the effects of tumoral IFNβ on macrophages, we conducted bulk RNA sequencing of M2-like polarized BMDM treated with Dox-induced YUMM5.2 Ifnb1eGFP supernatant, rIFNβ or controls as above. Expression profiles clustered depending on IFNβ-exposure irrespective of the IFNβ source by principal component analysis (Fig. 4A). Likewise, correlation analysis of log_2_-transformed mean gene counts of M2-like BMDM treated with supernatant from Dox-treated YUMM5.2 Ifnb1eGFP cells or rIFNβ demonstrated that neither tumor cell metabolites nor Dox residues significantly contributed to data variation (Fig. 4B). Thus, for subsequent analyses we focused on comparing the transcriptomes of BMDM treated with supernatant from Dox-treated YUMM5.2 Ifnb1eGFP cells to BMDM treated with supernatant from Dox-treated YUMM5.2 eGFP control cells. In order to comprehensively assess IFNβ-induced changes in macrophage polarization beyond our flow cytometric marker panel (Fig. 2C and D), we conducted GSEA of a curated literature-based gene set of 142 genes associated with the proinflammatory M1-like phenotype (Supplementary Table S3), which showed concordant upregulation in BMDM treated with supernatant from Dox-treated YUMM5.2 Ifnb1eGFP cells (Fig. 4C). Moreover, unsupervised GSEA of Molecular Signature Database Hallmark gene sets also showed induced expression of gene sets associated with proinflammatory immune responses such as interferon alpha response, interferon gamma response, allograft rejection, Il6 Jak Stat3 signaling and inflammatory response (Fig. 4D). Conversely, Hallmark gene sets associated with an anti-inflammatory and M2-like metabolic phenotype (28) such as fatty acid metabolism and cholesterol homeostasis, were suppressed in BMDM treated with supernatant from Dox-treated YUMM5.2 Ifnb1eGFP cells (Fig. 4D). Overall, these results demonstrate a proinflammatory M1-like re-education of M2-like macrophages following exposure to tumoral IFNβ. A subsequent DGE analysis of immune-related genes, curated based on literature, among all upregulated genes revealed macrophage activation markers such as *Aif1* (29) and *Cd69* (30), genes involved in immune cell infiltration, antigen presentation e.g. *H2-Q6* (*MHCI*) and T-cell activation such as *Cd86*, whose enhanced expression we had demonstrated in the Dox-induced YUMM5.2 Ifnb1eGFP TME (Fig 2C; Supplementary Fig. S3E) in IFNβ-exposed BMDMs (Fig. 4E). Collectively, we identified a myeloid type I IFN-response signature involved in mediating an effective antitumor immune response. To confirm that the myeloid type I IFN-response signature reflected the effects of tumoral IFNβ on TAM in our intracranial YUMM5.2 Ifnb1eGFP mouse model, we performed quantitative reverse transcription PCR on TAMs isolated from established YUMM5.2 Ifnb1eGFP tumors. Indeed, tumoral IFNβ induced by Dox diet significantly increased RNA expression of CD69 and MHCI by TAM (Fig. 4F and G). Next, we validated this altered transcriptional gene expression on protein level via immunohistochemistry staining on tissue sections of established YUMM5.2 Ifnb1eGFP tumors. CD69 (Fig. 4H) and MHCI (Fig. 4I) protein levels were higher in TAM of animals receiving Dox diet compared to TAMs of animals receiving control diet. Taken together, these results demonstrate that IFNβ secreted by tumor cells is sufficient to repolarize immunoregulatory TAM into proinflammatory macrophages inside the MBM TME by inducing a myeloid type I IFN-response signature.

### Myeloid type I IFN-response signature associates with an increased M1/M2 ratio and extended overall survival following radiotherapy in patients with MBM

**Figure 5:**
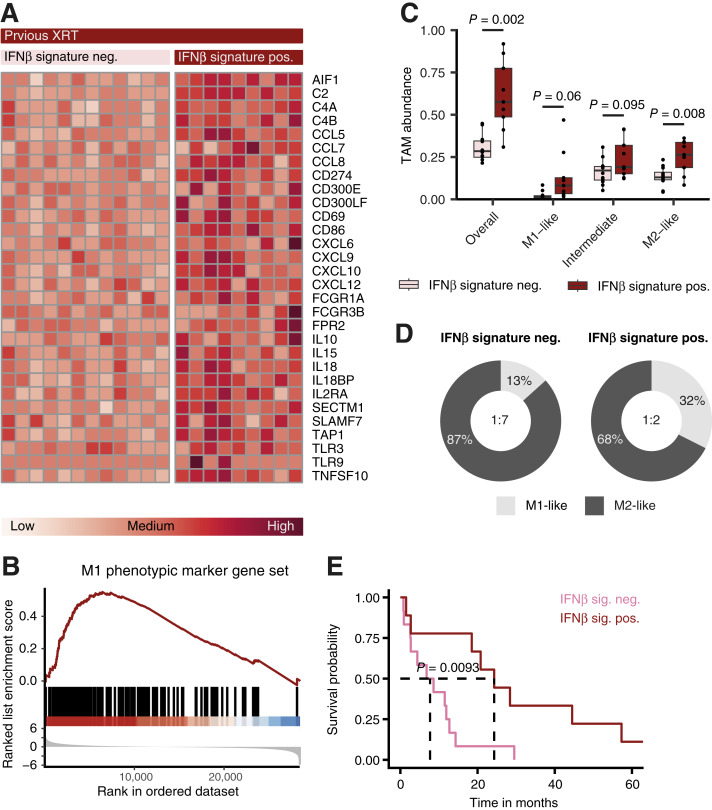
Irradiation induces the proinflammatory type I IFN-response signature in TAM associated with increased overall survival in patients with melanoma brain metastases. **A,** Heatmap of normalized gene counts of the myeloid type I IFN-response signature in previously irradiated MBM not expressing the myeloid type I IFN-response signature (neg., negative) compared to MBM expressing the myeloid type I IFN-response signature (pos., positive; *n* = 12 and *n* = 9). **B,** GSEA of a curated M1 phenotypic marker gene set in MBM from non-irradiated patients compared to MBM from previously irradiated patients. Enrichment Score = 0.55, NES = 2.06, adjusted *P* value = 1.9e–8 (*n* = 48 and *n* = 21). **C** and **D,** Deconvolution of TAM abundances in previously irradiated MBM not expressing the myeloid type I IFN-response signature (neg.) compared to MBM expressing the myeloid type I IFN-response signature (pos.) using CIBERSORTx (*n* = 12 and *n* = 9). Displayed as absolute proportion of each cell type in each sample in **C** or mean percentage per group and M1/M2 ratio in **D**. **E,** Kaplan–Meier survival estimates for the association of type I IFN-signature expression with the overall survival of irradiated MBM patients (*n* = 12 and *n* = 9). For the M1 phenotypic marker gene set see Supplementary Table S3. Data are expressed as individual values for **A**, median + IQR and individual values for **C** and mean for **D**. Statistical significance was determined by paired *t* test for **C** and log-rank test for **E**.

We next sought to probe the established myeloid type I IFN-response signature in post radiotherapy MBM by demonstrating IFNβ-mediated proinflammatory repolarization of TAM in these tumors. Radiotherapy is a type I IFN-inducing immunogenic anticancer therapy and a common treatment for patients presenting with multiple or difficult to access MBM (31). In a recently published bulk RNA sequencing data set of 48 radiotherapy-naïve and 21 previously irradiated MBM (23), we were able to show upregulation of the myeloid type I IFN-response signature in 9 out of 21 (43%) irradiated MBM (Fig. 5A; Supplementary Fig. S4A). Moreover, differential gene expression analysis revealed an overlap of 14 out of 53 (26%) genes from associated pathways and effector functions upregulated in irradiated MBM compared to radiotherapy-naïve MBM with DEGs in IFNβ-treated BMDM (Supplementary Fig. S4B), suggestive of type I IFN-signaling post-irradiation. To assess corresponding polarization changes in MBM-associated macrophages following radiotherapy, we performed GSEA of the literature-based curated M1-like marker gene set which showed enrichment in irradiated MBMs (Fig. 5B). Computational deconvolution of TAM polarization states using CIBERSORTx (32) however, showed no significantly higher abundance of M1-like TAM in the TME of type I IFN-response signature positive and previously irradiated MBM compared to signature negative irradiated MBM (Fig. 5C). Although overall TAM infiltration and abundance of M2-like TAM was significantly higher in irradiated and type I IFN-response signature positive MBM, the ratio of antitumor and immune-stimulatory M1-like to protumor and immune-regulatory M2-like TAM was markedly increased in these MBM compared to type I IFN-response signature negative MBM (Fig. 5D). Since a high M1/M2-like ratio has been shown to positively correlate with improved patient prognosis in other cancer types (33), we performed Kaplan–Meier survival estimates to analyze the association of a positive type I IFN-signature with the overall survival of patients with MBM following radiotherapy. Expression of the type I IFN-response signature was significantly associated with a prolonged overall survival post-irradiation (Fig. 5E; Supplementary Table S4). Overall, these findings suggest that while radiotherapy demonstrably induces a myeloid type I IFN response in some patients with MBM, it also signals through a range of other immune-stimulatory cytokines which influence MBM-associated macrophage polarization states, supporting our approach of studying the isolated effects of type I IFN on TAM using an inducible IFNβ-overexpressing mouse model.

## Discussion

In this study, we show that tumoral IFNβ induces a proinflammatory M1-like TAM phenotype and can functionally re-educate TAM in two MBM mouse models. Previous studies have highlighted the role of autocrine type I IFN signaling in sustaining an M1-like phenotype in the U-937 monocytic cell line (15). However, to our knowledge, the effects of paracrine tumoral IFNβ signaling on M2-like TAM and their potential re-polarization have not yet been reported. Type I IFN are frequently associated with immunogenic anticancer therapies such as radiotherapy, chemotherapy, and immunotherapy and essential for their efficacy (34). In a preclinical model of ovarian cancer as well as in a clinical trial for patients presenting with immunologically cold tumors (NCT03728179), type I IFN were shown to be associated with immune cell infiltration and response to immune checkpoint blockade following low-dose radiotherapy (35). Similar effects were described in a murine model of pancreatic cancer, where low-dose radiotherapy led to an increase in tumoricidal M1-like TAM characterized by inducible nitric oxide synthase (iNOS) expression, which mediated TIL recruitment and tumor rejection (36). These studies demonstrate that the observed immune-stimulatory effects of IFNβ not only apply to melanoma but to a broad range of solid tumors.

By making use of inducible IFNβ-overexpressing intracranial melanoma mouse models, we were able to eliminate confounding effects of immunogenic cell death, antigen and nucleic acid release and release of other immune-stimulatory cytokines associated with type I IFN–inducing anticancer therapies on TAM phenotype and functionality. Both the YUMM5.2 Ifnb1eGFP and B16-F10 Ifnb1eGFP cell lines showed reliant Dox-induced IFNβ-secretion both *in vitro* and *in vivo* (Figs. 1A–E, 3A and B) and *in vivo* Ifnb1eGFP tumor cell–associated macrophage phenotypes could be accurately reproduced using the *in vitro* BMDM system including INFβ exposure released from induced tumor cells and by means of recombinant protein (Figs. 2G–L, 3H and I). However, our models could only in part replicate the type I IFN–associated changes of TAM polarization states in patients with previously irradiated MBM (Fig. 5C). Whether these differences derive from murine-specific properties or from aforementioned confounding factors associated with radiotherapy remains to be elucidated. Of note, in both models, induction of tumoral IFNβ was reflected by clinical response as it led to a reduction in tumor growth (Figs. 1F and G, 3G), although IFNβ-dependent re-education of the TME differed slightly between the models. Particularly T cells seemed to be more prone to be affected by tumoral IFNβ release signified by increased infiltration, activation and proliferation of CD8^+^ and CD4^+^ T cells, respectively, in B16-F10 than in YUMM5.2 Ifnb1eGFP TMEs (Fig. 3D–F; Supplementary Fig. S3C). These differences are most likely to be attributable to distinct mutational landscapes of selected cell lines or growth and immune response dynamics which shape the TME and which may have not been detectable in the endpoint analysis of the YUMM5.2 TME. This hypothesis is underlined by our *in vitro* findings of BMDM-driven T-cell activation after pretreatment of BMDM with YUMM5.2 Ifnb1eGFP-derived IFNβ (Fig. 2J and K).

In this study, we identified a myeloid type I IFN-response signature comprising 30 genes and serving as a surrogate marker for transitory interferon expression in the context of type I IFN–inducing anticancer therapies (Fig. 4E). Three of these genes, i.e. *Fcgr1*, *Fcgr4* and *Tnfsf10*, also exhibit cytotoxic effector functions. The Fc receptors IgG Fcgr1 and Fcgr4 can induce antibody-dependent cell-mediated cytotoxicity and have been found to be essential for the antitumor activity of immunotherapies targeting both tumor-specific antigens as well as inhibitory immune-checkpoint proteins (37–39). While Fcgr1 and Fcgr4 rely on antibody binding for their tumoricidal activity, tumor necrosis factor-related apoptosis-inducing ligand Tnfsf10, also known as Trail, can directly induce apoptosis selectively in tumor cells (40). Tnfsf10 expression has been shown to positively correlate with overall survival, recurrence-free survival and immunotherapy response of patients with melanoma (41). In a cohort of previously irradiated patients with MBM, we were able to show upregulation of this myeloid type I IFN-response signature which associated with a favorable increase in the ratio of M1/M2-like TAM as well as extended overall survival, demonstrating clinical relevance of this radiation-induced re-education of TAM (Fig. 5E). Clinical data, in particular time between irradiation treatment and resection, did not significantly differ between MBM expressing (*n* = 9, 43%) and MBM not-expressing (*n* = 12, 57%) the myeloid type I IFN-response signature (Supplementary Table S4). However, type I IFN locus deletions (42) as well as recurrent epigenetic silencing of the STING signaling pathway, which is essential for type I IFN-induction following radiotherapy (43, 44), have been reported in melanoma and might account for the observed intertumoral heterogeneity. Although Xia and colleagues (44) attribute type I IFN-expression during interferon-inducing anticancer therapies to the tumoral compartment, we cannot exclude contribution of other cell types to type I IFN-secretion following irradiation in this cohort based on the bulk RNA sequencing data at hand, especially as myeloid cells themselves, but also other immune and stromal cells, are able to secrete type I IFN. It is tempting to speculate that this may contribute to some of the differences in TAM gene expression and polarization observed in our murine models.

Overall, our findings warrant further developments to identify surrogate markers of type I IFN signaling aberrations in these difficult-to-biopsy tumors. These markers could help stratify patients according to their type I IFN status and be predictive for response to irradiation treatment. This might be especially useful considering the high risk for radiation necrosis associated with stereotactic radiotherapy in patients with MBM, a detrimental adverse effect (45, 46). In addition to helping guide clinical decision making, increased serum IFNβ levels were found to be a strong predictor of early treatment response to combined radiotherapy and immunotherapy (CRI) in a clinical phase II study in patients with NSCLC brain metastases (NCT02221739) and were thought to contribute to the observed synergistic effects of these treatment modalities (27). Although recent studies on CRI demonstrated better local response rates and lower rates of local recurrence in patients with MBM compared to immunotherapy alone (47–49), the exact mechanisms underlying this synergy remain to be investigated. Our work provides insight into one possible mechanism linking radiation-induced type I IFN secretion to a proinflammatory M1-like MBM-associated macrophage phenotype, promoting an effective antitumor immune response. This hypothesis is supported by clinical data highlighting the importance of timing radiotherapy concomitantly to immunotherapy in CRI regimens (49, 50). Further preclinical studies using the inducible IFNβ-overexpressing YUMM5.2 and B16-F10 Ifnb1eGFP models will be necessary to characterize the effects of re-educated M1-like TAM on the efficacy of concomitant immune checkpoint inhibition and additional clinical trials on type I IFN–dependent anticancer therapies in MBM with larger case numbers, shorter intervals between treatment and resection as well as the use of single-cell technologies will be needed.

In conclusion, our work highlights the immune-stimulatory capacity of tumoral IFNβ in the context of type I IFN–inducing anticancer therapies. By re-educating intratumoral macrophages toward a proinflammatory M1-like phenotype, type I IFNs could help prime the TME and thereby increase response rates to subsequent immunotherapy in patients with MBM.

## Supplementary Material

Supplementary Figure S1 Supplementary Figure S1 characterizes modified cell lines, showing transduction vectors, confirmatory flow cytometry of protein induction, and in vitro proliferation.

Supplementary Figure S2 Supplementary Figure S2 depicts flow cytometry gating strategies for all assays.

Supplementary Figure S3 Supplementary Figure S3 shows characterization of intracranial tumor microenvironment and macrophages after IFN-beta exposure.

Supplementary Figure S4 Supplementary Figure S4 shows type I IFN response signature gene expression in human melanoma brain metastasis macrophages.

Supplementary Table S1 Supplementary Table S1 lists primers used.

Supplementary Table S2 Supplementary Table S2 lists antibodies and dyes used.

Supplementary Table S3 Supplementary Table S3 lists all genes included in the M1 phenotypic marker gene set, including respective references.

Supplementary Table S4 Supplementary Table S4 depicts MBM patient characteristics.
