# Supplementary material for: Tumoral Interferon Beta Induces an Immune-Stimulatory Phenotype in Tumor-Associated Macrophages in Melanoma Brain Metastases
Source: Cancer Res Commun. 2024 Aug 21;4(8):2189–202. doi: 10.1158/2767-9764.CRC-24-0024 (PMC11337092; doi:10.1158/2767-9764.CRC-24-0024)
Supplement: Supplementary Figure S1 — characterizes modified cell lines, showing transduction vectors, confirmatory flow cytometry of protein induction, and in vitro proliferation. [file crc-24-0024_supplementary_figure_s1_supps1.pdf]

Supplementary Figure S1

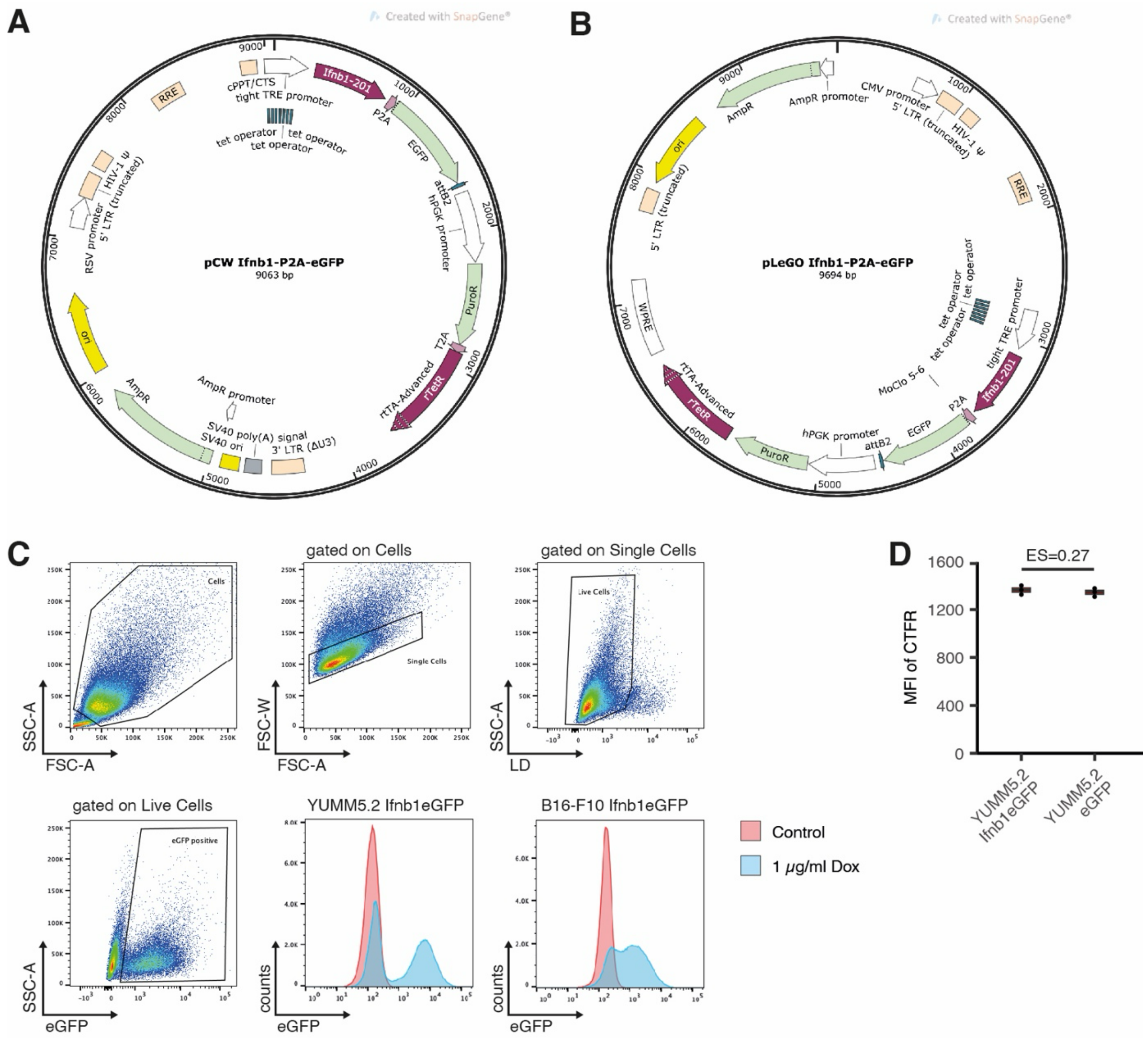

Supplementary Figure S1 Transduction of YUMM5.2 with pCW Ifnb1-P2A-eGFP and B16-F10 with pLeGO Ifnb1-P2A-eGFP and cell proliferation. A Vector map of lentiviral pCW Ifnb1-P2A-eGFP. **B** Vector map of lentiviral pLeGO Ifnb1-P2A-eGFP. **C** Gating strategy for YUMM5.2 Ifnb1eGFP and for B16-F10 Ifnb1eGFP cell sorting. Transduced YUMM5.2 Ifnb1eGFP and B16-F10 Ifnb1eGFP cells were treated with 1 µg/ml Dox or left untreated for 24 hours before cells were harvested and analyzed using flow cytometry. LD, live/dead marker. Cells with MFI > 10^3 were selected and used for subsequent assays. **D** Quantitative flow cytometry analysis of CTFR intensity in eGFP-positive tumor cells after 48 hours of treatment with 1 µg/ml Dox. Displayed as mean fluorescence intensity. (n=3) Statistical significance was determined by Mann-Whitney U test for **D**. ES, effect size.
