# Supplementary material for: Tumoral Interferon Beta Induces an Immune-Stimulatory Phenotype in Tumor-Associated Macrophages in Melanoma Brain Metastases
Source: Cancer Res Commun. 2024 Aug 21;4(8):2189–202. doi: 10.1158/2767-9764.CRC-24-0024 (PMC11337092; doi:10.1158/2767-9764.CRC-24-0024)
Supplement: Supplementary Figure S2 — depicts flow cytometry gating strategies for all assays. [file crc-24-0024_supplementary_figure_s2_supps2.pdf]

Supplementary Figure S2

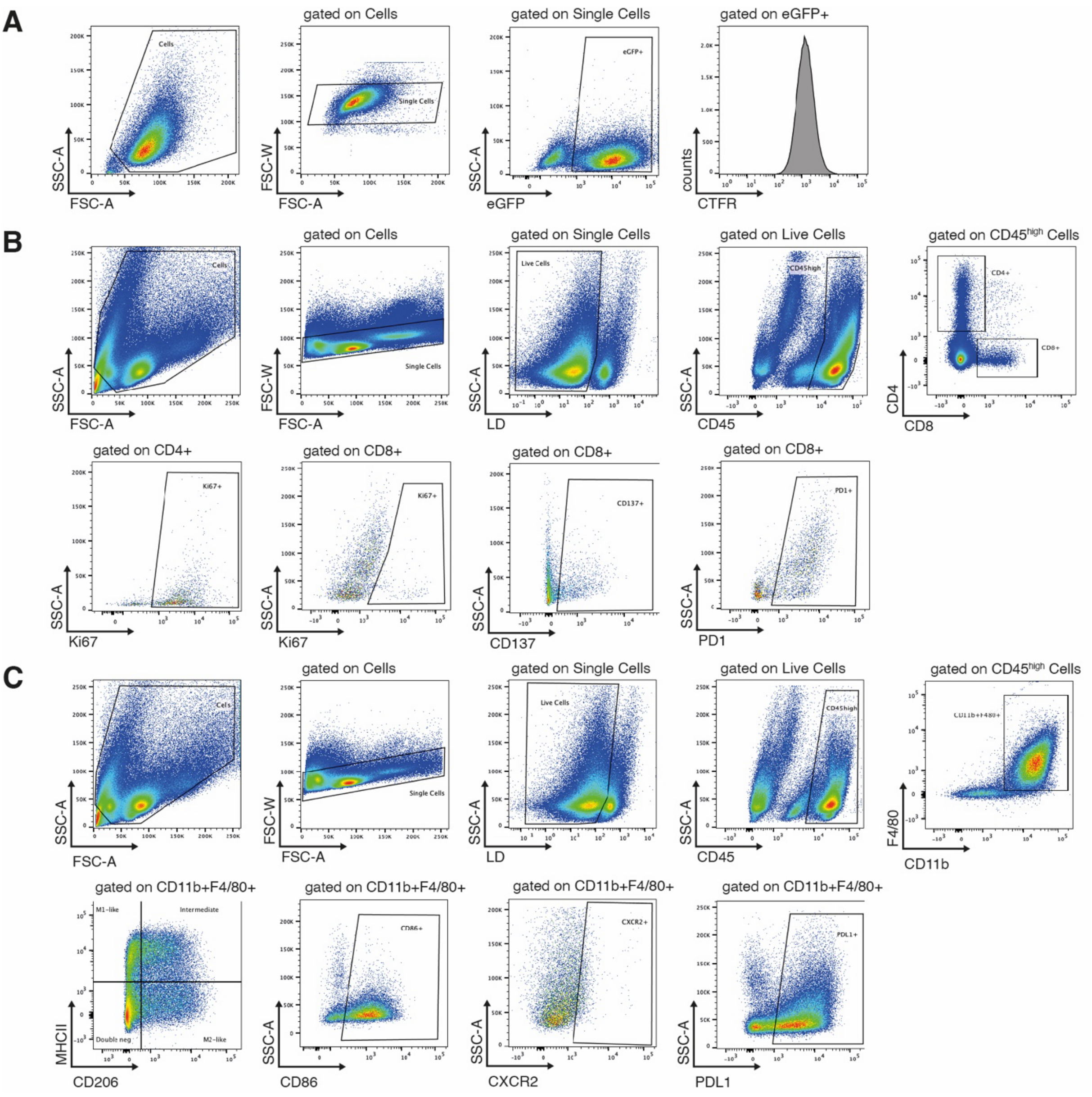

Supplementary Figure S2 Gating strategies for flow cytometry. A Gating strategy for *in vitro* proliferation assay. **B** Gating strategy for *in vivo* tumor-infiltrating T cell populations. **C** Gating strategy for *in vivo* TAM populations and *in vitro* IFNβ-treated BMDMs. For the analysis of TAM polarization states in the B16-F10 Ifnb1eGFP tumor model, CD206 and MHCII were also separately plotted against SSC-A.
