# Supplementary material for: Tumoral Interferon Beta Induces an Immune-Stimulatory Phenotype in Tumor-Associated Macrophages in Melanoma Brain Metastases
Source: Cancer Res Commun. 2024 Aug 21;4(8):2189–202. doi: 10.1158/2767-9764.CRC-24-0024 (PMC11337092; doi:10.1158/2767-9764.CRC-24-0024)
Supplement: Supplementary Figure S3 — shows characterization of intracranial tumor microenvironment and macrophages after IFN-beta exposure. [file crc-24-0024_supplementary_figure_s3_supps3.pdf]

Supplementary Figure S3

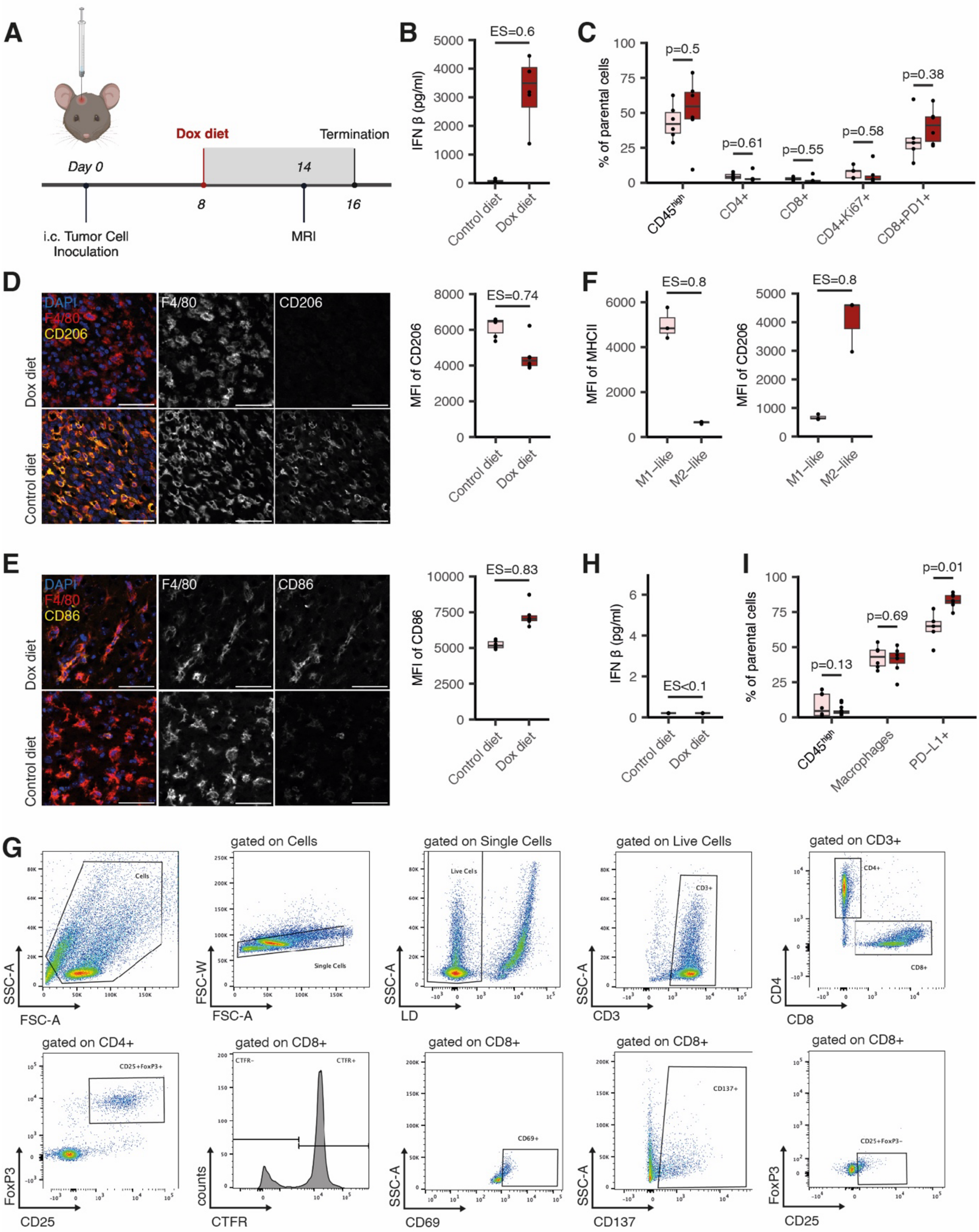

Supplementary Figure S3 Phenotypic and functional characterization of the tumor microenvironment and IFNβ-treated bone marrow-derived macrophages. A C57Bl/6J mice received control diet or Dox diet from day 8 on following intracranial YUMM5.2 Ifnb1eGFP tumor cell inoculation. Tumor growth was monitored by MRI on day 14 and tumors were excised on day 16. Created with BioRender.com. Related to Figure 2A-E. **B** Intratumoral IFNβ concentrations were analyzed by ELISA and are displayed in pg/ml. (n=6) **C** Quantitative flow

cytometry analysis of YUMM5.2 tumor-infiltrating T cell populations after control diet (light pink) or Dox diet (dark red). Displayed as percentage of parental population. (n=6) **D,E** Immunohistochemistry staining of F4/80 (red) and CD206 (**D**) and CD86 (**E**) (yellow) on tissue sections from mice with intracranial YUMM5.2 Ifnb1eGFP tumors after control diet or Dox diet. Left, representative images. Scale bars represent 50 µm. Right, quantitative analysis of CD206 (**D**) and CD86 (**E**) expression in F4/80+ cells. Displayed as mean fluorescence intensity. (n=3, 2 fields of view per animal). **F** Quantitative flow cytometry analysis of MHCII and CD206 in bone marrow-derived and either M1-like or M2-like polarized macrophages after 48 hours of polarization treatment as quality control. Displayed as mean fluorescence intensity. (n=3) **G** Gating strategy for *in vitro* mixed-leukocyte-reaction assay**. H** Blood was drawn from mice with intracranial B16-F10 Ifnb1eGFP tumors after 15 days of control diet or Dox diet. Plasma IFNβ concentrations were analyzed by ELISA and are displayed in pg/ml. (n=8) **I** Quantitative flow cytometry analysis of B16-F10 Ifnb1eGFP TAMs after 15 days of control diet (light pink) or Dox diet (dark red). Displayed as percentage of parental population. (n=6 and n=7) For gating strategies see Supplementary Figure S2. Data are expressed as median + IQR and individual values for **B**, **C**, **D**, **E**, **F**, **H**, **I**. Statistical significance was determined by Mann-Whitney U test for **B**, **D**, **E**, **F**, **H** and by paired t test for **C**, **I**. ES, effect size.
