# Supplementary material for: Tumoral Interferon Beta Induces an Immune-Stimulatory Phenotype in Tumor-Associated Macrophages in Melanoma Brain Metastases
Source: Cancer Res Commun. 2024 Aug 21;4(8):2189–202. doi: 10.1158/2767-9764.CRC-24-0024 (PMC11337092; doi:10.1158/2767-9764.CRC-24-0024)
Supplement: Supplementary Figure S4 — shows type I IFN response signature gene expression in human melanoma brain metastasis macrophages. [file crc-24-0024_supplementary_figure_s4_supps4.pdf]

Supplementary Figure S4

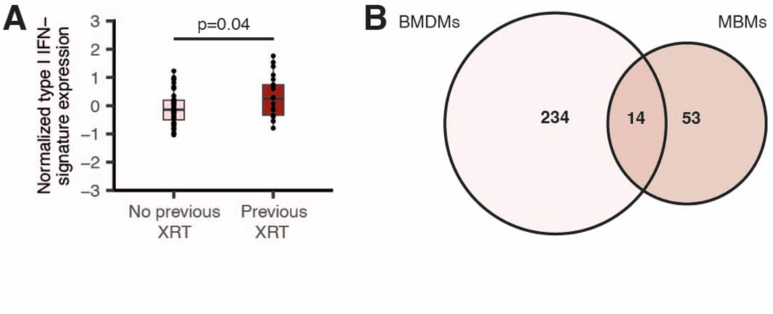

Supplementary Figure S4 Type I IFN-response signature in melanoma brain metastasis-associated macrophages. A Normalized expression of the myeloid type I IFN-response signature gene set in MBMs from non-irradiated patients compared to previously irradiated patients. (n=48 and n=21) **B** Venn diagram of overlapping DEGs from associated pathways and effector functions between IFNβ-treated bone marrow-derived macrophages (BMDMs) and irradiated MBM samples. Data are expressed as median + IQR and individual values for **A**. Statistical significance was determined by paired t test for **A**.
