# Supplementary material for: Tumoral Interferon Beta Induces an Immune-Stimulatory Phenotype in Tumor-Associated Macrophages in Melanoma Brain Metastases
Source: Cancer Res Commun. 2024 Aug 21;4(8):2189–202. doi: 10.1158/2767-9764.CRC-24-0024 (PMC11337092; doi:10.1158/2767-9764.CRC-24-0024)
Supplement: Supplementary Table S1 — lists primers used. [file crc-24-0024_supplementary_table_s1_supps1.pdf]

Supplementary Table S1

Target	Sequence
mIfnb1 forward	5’CAAAAAAGCAGGCTCCACCATGAACAACAGGTGGATCCTC
mIfnb1 reverse	5’CAAGAAAGCTGGGTTGTTTTGGAAGTTTCTGGTAAGTCTTC
mCd69 forward	5’AGCTACATCTCTCCGTGGAC
mCd69 reverse	5’TATACTGGTGCCATGGTCCTT
mMHCI (H2-Q6) forward	5’AGAAGTGGGCATCTGTGGTG
mMHCI (H2-Q6) reverse	5’ATGGAGGAGGCTCCCATCTC
mGapdh forward	5’GAAGGTCGGAGTCAACGGATTTG
mGapdh reverse	5’CATGTAAACCATGTAGTTGAGGT

Supplementary Table S1 Primers. Forward and reverse primers used for gene isolation from genomic DNA and for RT-qPCR.
