# Supplementary material for: Tumoral Interferon Beta Induces an Immune-Stimulatory Phenotype in Tumor-Associated Macrophages in Melanoma Brain Metastases
Source: Cancer Res Commun. 2024 Aug 21;4(8):2189–202. doi: 10.1158/2767-9764.CRC-24-0024 (PMC11337092; doi:10.1158/2767-9764.CRC-24-0024)
Supplement: Supplementary Table S2 — lists antibodies and dyes used. [file crc-24-0024_supplementary_table_s2_suppst2.pdf]

Supplementary Table S2

***FACS:* In vivo T cell subsets**				
**Target**	**Label**	**Clone**	**Supplier, Cat#, RRID**	**Dilution**
Fixable Viability Dye	eFluor780	-	ThermoFisher, Cat# 65-0865-14	1:1000
CD45	BV510	30-F11	Biolegend, Cat# 103137, RRID AB_2563061	1:100
CD8a	AF700	53-6.7	Biolegend, Cat# 100730, RRID AB_493703	1:100
CD4	BV421	RM4-5	Biolegend, Cat# 100543, RRID AB_2563052	1:100
Ki67	BV605	16A8	Biolegend, Cat# 652413, RRID AB_2562664	1:100
CD137	PE-Cy7	17B5	ThermoFisher, Cat# 25-1371-82, RRID AB_2573398	1:100
PD1	APC	RMP1	Biolegend, Cat# 109112, RRID AB_10612938	1:100
***FACS:* In vivo myeloid subsets and bone marrow-derived macrophages**				
**Target**	**Label**	**Clone**	**Supplier, Cat#, RRID**	**Dilution**
Fixable Viability Dye	eFluor780	-	ThermoFisher, Cat# 65-0865-14	1:1000
CD45	BV510	30-F11	Biolegend, Cat# 103137, RRID AB_2563061	1:100
CD11b	BV605	M1/70	Biolegend, Cat# 101237, RRID AB_2565431	1:100
F4/80	BV421	BM8	Biolegend, Cat# 123132, RRID AB_2563102	1:100
I-A/I-E (MHCII)	AF700	M5/114.15.2	Biolegend, Cat# 107622, RRID AB_493727	1:100
CD206	PE-Cy7	C068C2	Biolegend, Cat# 141720, RRID AB_2562248	1:100
CD86	FITC	GL1	Biolegend, Cat# 105005, RRID AB_313149	1:200
CXCR2	PerCP-Cy5.5	SA044G4	Biolegend, Cat# 149307, RRID AB_2565695	1:200
PDL1	BV711	10F.9G2	Biolegend, Cat# 124319, RRID AB_2563619	1:100
***FACS:* Mixed-leukocyte-reaction assay**				
**Target**	**Label**	**Clone**	**Supplier, Cat#, RRID**	**Dilution**
Fixable Viability Dye	eFluor780	-	ThermoFisher, Cat# 65-0865-14	1:1000
CD3	FITC	17A2	Biolegend, Cat# 100203, RRID AB_312660	1:50
CD8a	Pe-Cy7	53-6.1	ThermoFisher, Cat# 25-0081, RRID AB_469584	1:200
CD4	PB	GK1.5	Biolegend, Cat# 100428, RRID AB_493647	1:800
CD25	PerCP-Cy5.5	3C7	Biolegend, Cat# 101912, RRID AB_10613642	1:200
FoxP3	PE	MF-14	Biolegend, Cat# 126404, RRID AB_1089118	1:200
CTFR	APC	-	ThermoFisher, Cat# C34564	-
CD69	BV605	Hi.2F3	Biolegend, Cat# 104529, RRID AB_2563062	1:100
CD137	PE-Cy7	17B5	ThermoFisher, Cat# 25-1371-82, RRID AB_2573398	1:100
Immunohistochemistry:				
**Target**	**Host species**	**Clone**	**Supplier, Cat#, RRID**	**Dilution**
F4/80	Rabbit	D4C8V	Cell Signaling, Cat# 30325S, RRID AB_2798990	1:100
CD69	Armenian hamster	H1.2F3	ThermoFisher, Cat# 14-0691-82, RRID AB_467325	1:100
MHCI	Rat	R1-21.2	Abcam, Cat# ab281904, RRID AB_3097724	1:100
CD206	Rat	MR5D3	ThermoFisher, Cat# MA5-16871, RRID AB_2538349	1:200
CD86	Rat	GL1	ThermoFisher, Cat# 14-0862-81, RRID AB_467367	1:200

Supplementary Table S2 Antibodies and dyes. Antibodies and dyes used for quantitative flow cytometry analysis and immunohistochemistry.
