# Supplementary material for: Tumoral Interferon Beta Induces an Immune-Stimulatory Phenotype in Tumor-Associated Macrophages in Melanoma Brain Metastases
Source: Cancer Res Commun. 2024 Aug 21;4(8):2189–202. doi: 10.1158/2767-9764.CRC-24-0024 (PMC11337092; doi:10.1158/2767-9764.CRC-24-0024)
Supplement: Supplementary Table S3 — lists all genes included in the M1 phenotypic marker gene set, including respective references. [file crc-24-0024_supplementary_table_s3_suppst3.pdf]

Supplementary Table S3

No.	Gene	Reference
1	Stat1	Orecchioni, M. et al. (2019), (1)
2	Nos2	Orecchioni, M. et al. (2019), (1)
3	Cd86	Orecchioni, M. et al. (2019), (1)
4	Cd40	Orecchioni, M. et al. (2019), (1)
5	Ptgs2	Orecchioni, M. et al. (2019), (1)
6	Il12a	Orecchioni, M. et al. (2019), (1)
7	Il12b	Orecchioni, M. et al. (2019), (1)
8	Icam1	Orecchioni, M. et al. (2019), (1)
9	Ifit2	Orecchioni, M. et al. (2019), (1)
10	Vcam1	Orecchioni, M. et al. (2019), (1)
11	Rsad2	Orecchioni, M. et al. (2019), (1)
12	Gpr84	Orecchioni, M. et al. (2019), (1)
13	Nfkbiz	Orecchioni, M. et al. (2019), (1)
14	Ehd1	Orecchioni, M. et al. (2019), (1)
15	Traf1	Orecchioni, M. et al. (2019), (1)
16	Ccnd2	Orecchioni, M. et al. (2019), (1)
17	Socs3	Orecchioni, M. et al. (2019), (1)
18	Adora2a	Orecchioni, M. et al. (2019), (1)
19	Serpine1	Orecchioni, M. et al. (2019), (1)
20	Serpinb2	Orecchioni, M. et al. (2019), (1)
21	Slfn1	Orecchioni, M. et al. (2019), (1)
22	Cd38	Orecchioni, M. et al. (2019), (1)
23	Inhba	Orecchioni, M. et al. (2019), (1)
24	Lcn2	Orecchioni, M. et al. (2019), (1)
25	Mefv	Orecchioni, M. et al. (2019), (1)
26	Nfkb2	Orecchioni, M. et al. (2019), (1)
27	AW112010	Orecchioni, M. et al. (2019), (1)
28	Il15ra	Orecchioni, M. et al. (2019), (1)
29	Irf7	Orecchioni, M. et al. (2019), (1)
30	Zbp1	Orecchioni, M. et al. (2019), (1)
31	Pilra	Orecchioni, M. et al. (2019), (1)
32	Oas3	Orecchioni, M. et al. (2019), (1)
33	Ifih1	Orecchioni, M. et al. (2019), (1)
34	Slco3a1	Orecchioni, M. et al. (2019), (1)
35	Nfkbia	Orecchioni, M. et al. (2019), (1)
36	Met	Orecchioni, M. et al. (2019), (1)
37	Isg20	Orecchioni, M. et al. (2019), (1)
38	Stat2	Orecchioni, M. et al. (2019), (1)
39	Batf2	Orecchioni, M. et al. (2019), (1)
40	Tlr2	Orecchioni, M. et al. (2019), (1)
41	Cflar	Orecchioni, M. et al. (2019), (1)
42	Hdc	Orecchioni, M. et al. (2019), (1)
43	Nupr1	Orecchioni, M. et al. (2019), (1)
44	Igtp	Orecchioni, M. et al. (2019), (1)
45	Gas7	Orecchioni, M. et al. (2019), (1)
46	Mmp14	Orecchioni, M. et al. (2019), (1)
47	Dhx58	Orecchioni, M. et al. (2019), (1)
48	Agrn	Orecchioni, M. et al. (2019), (1)
49	Vcan	Orecchioni, M. et al. (2019), (1)
50	Ifi35	Orecchioni, M. et al. (2019), (1)
51	Jak2	Orecchioni, M. et al. (2019), (1)
52	Arg2	Orecchioni, M. et al. (2019), (1)
53	Gch1	Orecchioni, M. et al. (2019), (1)
54	Peli1	Orecchioni, M. et al. (2019), (1)
55	Ddx60	Orecchioni, M. et al. (2019), (1)
56	Vasp	Orecchioni, M. et al. (2019), (1)
57	Zufsp	Orecchioni, M. et al. (2019), (1)
58	Eif2ak2	Orecchioni, M. et al. (2019), (1)

59	Sod2	Orecchioni, M. et al. (2019), (1)
60	Cd274	Orecchioni, M. et al. (2019), (1)
61	Ell2	Orecchioni, M. et al. (2019), (1)
62	Ptges	Orecchioni, M. et al. (2019), (1)
63	Pvr	Orecchioni, M. et al. (2019), (1)
64	Batf	Orecchioni, M. et al. (2019), (1)
65	Cxcl9	Orecchioni, M. et al. (2019), (1)
66	Slc25a37	Orecchioni, M. et al. (2019), (1)
67	Ms4a6c	Orecchioni, M. et al. (2019), (1)
68	Pstpip2	Orecchioni, M. et al. (2019), (1)
69	Itgal	Orecchioni, M. et al. (2019), (1)
70	Parp9	Orecchioni, M. et al. (2019), (1)
71	Notch1	Orecchioni, M. et al. (2019), (1)
72	Hck	Orecchioni, M. et al. (2019), (1)
73	Snx20	Orecchioni, M. et al. (2019), (1)
74	Nfkbib	Orecchioni, M. et al. (2019), (1)
75	Ccrl2	Orecchioni, M. et al. (2019), (1)
76	Samsn1	Orecchioni, M. et al. (2019), (1)
77	Dusp2	Orecchioni, M. et al. (2019), (1)
78	Cd14	Orecchioni, M. et al. (2019), (1)
79	Cxcl16	Orecchioni, M. et al. (2019), (1)
80	Slfn2	Orecchioni, M. et al. (2019), (1)
81	Jdp2	Orecchioni, M. et al. (2019), (1)
82	Tapbpl	Orecchioni, M. et al. (2019), (1)
83	Stat1	Orecchioni, M. et al. (2019), (1)
84	Rbpms	Orecchioni, M. et al. (2019), (1)
85	Rab32	Orecchioni, M. et al. (2019), (1)
86	Map3k5	Orecchioni, M. et al. (2019), (1)
87	Il1rn	Orecchioni, M. et al. (2019), (1)
88	Flnb	Orecchioni, M. et al. (2019), (1)
89	Birc3	Orecchioni, M. et al. (2019), (1)
90	Tap2	Orecchioni, M. et al. (2019), (1)
91	Mitd1	Orecchioni, M. et al. (2019), (1)
92	Ggct	Orecchioni, M. et al. (2019), (1)
93	Tnip1	Orecchioni, M. et al. (2019), (1)
94	Cd86	Orecchioni, M. et al. (2019), (1)
95	Irf9	Orecchioni, M. et al. (2019), (1)
96	Il17ra	Orecchioni, M. et al. (2019), (1)
97	Trim25	Orecchioni, M. et al. (2019), (1)
98	Lmo4	Orecchioni, M. et al. (2019), (1)
99	Acsl1	Orecchioni, M. et al. (2019), (1)
100	Ebi3	Orecchioni, M. et al. (2019), (1)
101	Gramd1a	Orecchioni, M. et al. (2019), (1)
102	Itga5	Orecchioni, M. et al. (2019), (1)
103	Acp5	Orecchioni, M. et al. (2019), (1)
104	Rnf114	Orecchioni, M. et al. (2019), (1)
105	Agtrap	Orecchioni, M. et al. (2019), (1)
106	Psmb9	Orecchioni, M. et al. (2019), (1)
107	Ifnar2	Orecchioni, M. et al. (2019), (1)
108	St3gal3	Orecchioni, M. et al. (2019), (1)
109	Slc31a1	Orecchioni, M. et al. (2019), (1)
110	Tnfrsf1b	Orecchioni, M. et al. (2019), (1)
111	Mtdh	Orecchioni, M. et al. (2019), (1)
112	Cpd	Orecchioni, M. et al. (2019), (1)
113	Psmb8	Orecchioni, M. et al. (2019), (1)
114	Skil	Orecchioni, M. et al. (2019), (1)
115	Pdpn	Orecchioni, M. et al. (2019), (1)
116	Snx10	Orecchioni, M. et al. (2019), (1)
117	Syk	Orecchioni, M. et al. (2019), (1)
118	Denr	Orecchioni, M. et al. (2019), (1)
119	Gadd45b	Orecchioni, M. et al. (2019), (1)
120	Slc15a3	Orecchioni, M. et al. (2019), (1)

121	Irf1	Xie, C. et al. (2016), (2)
122	Irf5	Xie, C. et al. (2016), (2)
123	Ifnb1	Xie, C. et al. (2016), (2)
124	Il6	Xie, C. et al. (2016), (2)
125	Tnfaip3	Xie, C. et al. (2016), (2)
126	Il23	Günthner, R. and H.J. Anders (2013), (3)
127	Akt2	Boutilier, A.J. and S.F. Elsawa (2021), (4)
128	Shp-1	Boutilier, A.J. and S.F. Elsawa (2021), (4)
129	Ship	Boutilier, A.J. and S.F. Elsawa (2021), (4)
130	MyD88	Boutilier, A.J. and S.F. Elsawa (2021), (4)
131	Il1b	Boutilier, A.J. and S.F. Elsawa (2021), (4)
132	Cxcl2	Boutilier, A.J. and S.F. Elsawa (2021), (4)
133	Il1a	Boutilier, A.J. and S.F. Elsawa (2021), (4)
134	Il6	Boutilier, A.J. and S.F. Elsawa (2021), (4)
135	Tnf	Boutilier, A.J. and S.F. Elsawa (2021), (4)
136	Cxcl13	Boutilier, A.J. and S.F. Elsawa (2021), (4)
137	Marco	Boutilier, A.J. and S.F. Elsawa (2021), (4)
138	Ccl11	Wu, K. et al. (2020), (5)
139	Il23a	Wu, K. et al. (2020), (5)
140	Csf2	Wu, K. et al. (2020), (5)
141	Csf3	Wu, K. et al. (2020), (5)
142	Ccl1	Wu, K. et al. (2020), (5)

Supplementary Table S3 M1 phenotypic marker gene set. Curated literature-based gene set of 142 genes associated with the pro-inflammatory M1-like phenotype.

References:

1. Orecchioni M, Ghosheh Y, Pramod AB, Ley K. Macrophage Polarization: Different Gene Signatures in M1(LPS+) vs. Classically and M2(LPS-) vs. Alternatively Activated Macrophages. *Front Immunol.* **2019**;10:1084.
2. Xie C, Liu C, Wu B, Lin Y, Ma T, Xiong H, et al. Effects of IRF1 and IFN-β interaction on the M1 polarization of macrophages and its antitumor function. *International Journal of Molecular Medicine.* **2016**;38(1):148-60.
3. Günthner R, Anders HJ. Interferon-regulatory factors determine macrophage phenotype polarization. *Mediators Inflamm.* **2013**;2013:731023.
4. Boutilier AJ, Elsawa SF. Macrophage Polarization States in the Tumor Microenvironment. *International Journal of Molecular Sciences.* **2021**;22(13):6995.
5. Wu K, Lin K, Li X, Yuan X, Xu P, Ni P, Xu D. Redefining Tumor-Associated Macrophage Subpopulations and Functions in the Tumor Microenvironment. *Front Immunol.* **2020**;11:1731.
