# Supplementary material for: Tumoral Interferon Beta Induces an Immune-Stimulatory Phenotype in Tumor-Associated Macrophages in Melanoma Brain Metastases
Source: Cancer Res Commun. 2024 Aug 21;4(8):2189–202. doi: 10.1158/2767-9764.CRC-24-0024 (PMC11337092; doi:10.1158/2767-9764.CRC-24-0024)
Supplement: Supplementary Table S4 — depicts MBM patient characteristics. [file crc-24-0024_supplementary_table_s4_suppst4.pdf]

Supplementary Table S4

Myeloid type I IFN-response signature	n	Sex (n, %)		Age (years)			Irradiation regime (n, %)		Time between irradiation and surgery (months)			Overall survival (months)		
		F	M	Mean	SD	Range	SRS	WBRT	Mean	SD	Range	Mean	SD	Range
**Neg.**	12	5, 42	7, 58	51.4	15.2	26-76	9, 75	3, 25	7.2	7.4	0.2-27.8	8.9	8.1	0.8-29.5
**Pos.**	9	5, 56	4, 44	48.2	14.4	31-74	8, 88.9	1, 11.1	7.87	7.31	0.6-17.8	29.5	22.8	1.5-67.4

Supplementary Table S4 Melanoma brain metastasis patient characteristics. Clinical data of MBM patients. F=female, M=male, SD=standard deviation, SRS=stereotactic radiosurgery, WBRT=whole brain radiation therapy.
